# Target‐Directed Azide‐Alkyne Cycloaddition for Assembling HIV‐1 TAR RNA Binding Ligands

**DOI:** 10.1002/anie.202003461

**Published:** 2020-05-25

**Authors:** Rakesh Paul, Debasish Dutta, Raj Paul, Jyotirmayee Dash

**Affiliations:** ^1^ School of Chemical Sciences Indian Association for the Cultivation of Science Jadavpur Kolkata 700 032 India

**Keywords:** click chemistry, cycloaddition, HIV-1 TAR RNA, peptidomimetics, Tat peptide

## Abstract

The highly conserved HIV‐1 transactivation response element (TAR) binds to the trans‐activator protein Tat and facilitates viral replication in its latent state. The inhibition of Tat–TAR interactions by selectively targeting TAR RNA has been used as a strategy to develop potent antiviral agents. Therefore, HIV‐1 TAR RNA represents a paradigmatic system for therapeutic intervention. Herein, we have employed biotin‐tagged TAR RNA to assemble its own ligands from a pool of reactive azide and alkyne building blocks. To identify the binding sites and selectivity of the ligands, the in situ cycloaddition has been further performed using control nucleotide (TAR DNA and TAR RNA without bulge) templates. The hit triazole‐linked thiazole peptidomimetic products have been isolated from the biotin‐tagged target templates using streptavidin beads. The major triazole lead generated by the TAR RNA presumably binds in the bulge region, shows specificity for TAR RNA over TAR DNA, and inhibits Tat–TAR interactions.

## Graphical abstract

**Taking the lead**: The highly dynamic secondary TAR RNA structure of HIV‐1 is used to template cycloaddition reactions leading to the generation of triazole‐containing thiazole peptidomimetics as selective leads. The triazole ligand with flexible methylene units selectively binds to TAR RNA over TAR RNA without a bulge and TAR DNA. The ligand presumably binds in the bulge region of the TAR RNA and inhibits its interaction with the Tat peptide.

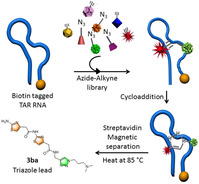

Human immunodeficiency virus type‐1 (HIV‐1)^[1]^ contains a cis‐acting regulatory element called TAR RNA located upstream of the transcriptional start site (+1 to +59).^[2]^ TAR RNA forms a stable hairpin structure with a hexanucleotide loop and a three base bulge (UCU). A trans‐activator protein Tat binds to the bulge region of the TAR RNA and activates HIV‐1 replication by stimulating the elongation efficiency of RNA pol II.^[3]^ The interaction between TAR and Tat is essential for viral replication and growth; however, mutated TAR shows a reduced affinity for Tat and is unable to replicate efficiently.^[4]^ Various strategies have been developed to inhibit viral replication, such as targeting the HIV‐1 protease and the long terminal repeat (LTR) region. Although numerous classes of small molecules have been reported,[^5^, ^6^] mutated virus makes them ineffective over time.^[7]^ However, the TAR sequence and its heterogeneity are highly conserved throughout the evolution of the virus (Figure 1 a).[^8^, ^9^] Therefore, the TAR RNA can be targeted for an anti‐HIV therapeutic strategy because of its structural integrity and central role in HIV‐1 replication.

**Figure 1:**
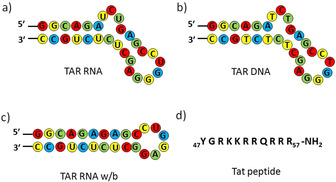
Sequences of a) target TAR RNA, b) TAR DNA, c) TAR RNA w/b, and d) Tat peptide used in the study.

Herein we demonstrate the rapid discovery of HIV‐1 TAR‐Tat inhibitors by TAR RNA guided in situ cycloaddition of azide and alkyne fragments. The in situ cycloaddition was also performed with a TAR RNA without the UCU bulge (TAR RNA w/b; w/b=without bulge) and a TAR DNA (Figure 1). The target‐guided synthetic (TGS) approach, commonly known as an in situ click reaction, accelerates the discovery of novel pharmaceutical molecules through the involvement of the biological target itself in the selection process.^[10]^ This bio‐orthogonal approach has been demonstrated previously using enzymes and a few nucleic acid targets;^[11]^ however, TAR RNA has not been employed as a target to date. The in situ click approach is a unique process which relies on target‐guided Huisgen 1,3‐dipolar cycloaddition of alkyne and azide residues to produce five‐membered nitrogen‐containing triazole heterocycles.^[12]^ Triazoles are stable under oxidative or reductive conditions as well as to basic or acidic hydrolysis. Furthermore, they actively participate in hydrogen bonding and π–π stacking interactions with biological targets.^[13]^

Both alkynes and azides are energetic species and there is a high kinetic barrier for cycloaddition. The kinetic barrier can be overcome by bringing the reactants into proximity with a biological template or with a catalyst.^[14]^ The biological target samples various combinations of the alkynes and azides and synthesizes the best binders with high efficacy. Given the dynamic folding topology of RNA structures, the in situ cycloaddition approach could be a potential strategy for generating selective RNA‐binding ligands. Various antibiotics and different classes of ligands have been reported previously for targeting TAR RNA.[^5^, ^6^] Herein, TAR RNA has been employed for the first time as a template to generate its specific binder that could efficiently inhibit Tat–TAR interactions.

For the in situ cycloaddition, we designed and synthesized a library of alkyne (**1 a**–**d**) and azide (**2 a**–**k**) building blocks (Figure 2 a, see also Schemes S1–S3 in the Supporting Information). Alkynes **1 a**–**c** consist of a different number of five‐membered thiazole heterocycles, prepared by iterative amide coupling. The thiazole ring system and peptides are privileged scaffolds in medicinal chemistry.^[15]^ Alkyne **1 d** is considered to have pharmacological activities because of its carbazole ring system.^[16]^ Our azide library **2 a**–**k** consists of aliphatic and aromatic functional groups such as amines (**2 a**, **2 e**, **2 f**, and **2 i**), a carboxylic acid (**2 b**), a nitro (**2 d**), an ester (**2 g**), an amino acid (**2 h**), a carboxamide (**2 j**), and a guanosine azide (**2 k**). The combinations of these building blocks could lead to 88 possible regioisomers (*anti*‐(1,4)‐ and *syn*‐(1,5)‐triazoles).

**Figure 2:**
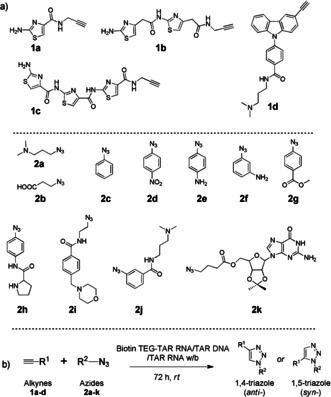
a) Structure of the thiazole alkynes (**1 a**–**c**), carbazole alkyne (**1 d**), and azides (**2 a**–**k**). b) Biotin TEG‐templated in situ cycloaddition of azide and alkyne fragments. TEG=triethyleneglycol.

The multicomponent reaction consisting of 4 alkynes and 11 azides was stirred in the presence of TAR RNA to generate potential hit ligands.^[17]^ As a model system for our study, we used a 5′‐biotin‐tagged 29 nt TAR RNA (Figure 1 a). The biotin‐tagged RNA can be easily separated from the reaction mixture along with the hit triazole products by magnetic separation using streptavidin magnetic beads (Figure 3). For the in situ RNA‐templated reaction, the alkynes and azides were incubated in a 1:4 ratio together with the target TAR RNA (10 μm) in 20 mm sodium cacodylate, 180 mm NaCl, and 10 mm MgCl_2_ buffer at pH 7.4 and 25 °C for 72 h. To identify the selective leads for the TAR RNA, control experiments were performed with biotinylated TAR RNA w/b and biotinylated TAR DNA (Figure 1 b,c).

**Figure 3:**
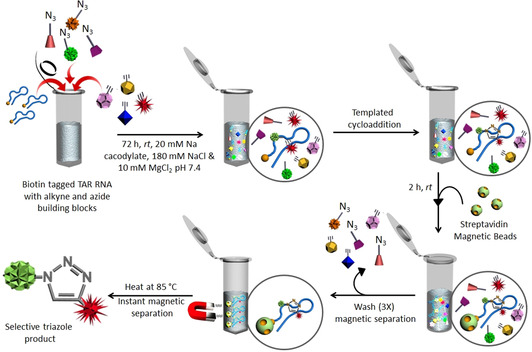
Schematic representation of the templated cycloaddition between selective azide and alkyne fragments using biotin‐tagged TAR RNA (for experimental details, see the Supporting Information).

The hit products were characterized by HPLC‐MS analysis, which showed the formation of two triazole products **3 ca** (cycloadduct of alkyne **1 c** and azide **2 a**) and **3 ba** (cycloadduct of alkyne **1 b** and azide **2 a**) in a ratio of 41:59 by the TAR RNA (Figure 4 a(i)). Since the triazole product **3 ca** was also formed in the presence of the control TAR DNA (Figure 4 a(ii), see also Figure S2a), the triazole lead **3 ba** was identified as the selective lead for TAR RNA. In addition, triazole product **3 aa** (cycloadduct of alkyne **1 a** and azide **2 a**) was selectively formed using the control TAR RNA w/b (Figure 4 a(iii)). The TAR RNA leads **3 ca** and **3 ba** were not formed by the TAR RNA w/b, thus suggesting an interaction between these ligands and the UCU bulge region of the TAR RNA. We further performed in situ cycloaddition of individual alkynes with the azide library in the presence of the TAR RNA and controls (Figure S2b). HPLC and ESI‐MS analysis showed the reactions yielded similar lead compounds (Figure 4 b, see also Figure S2a).

**Figure 4:**
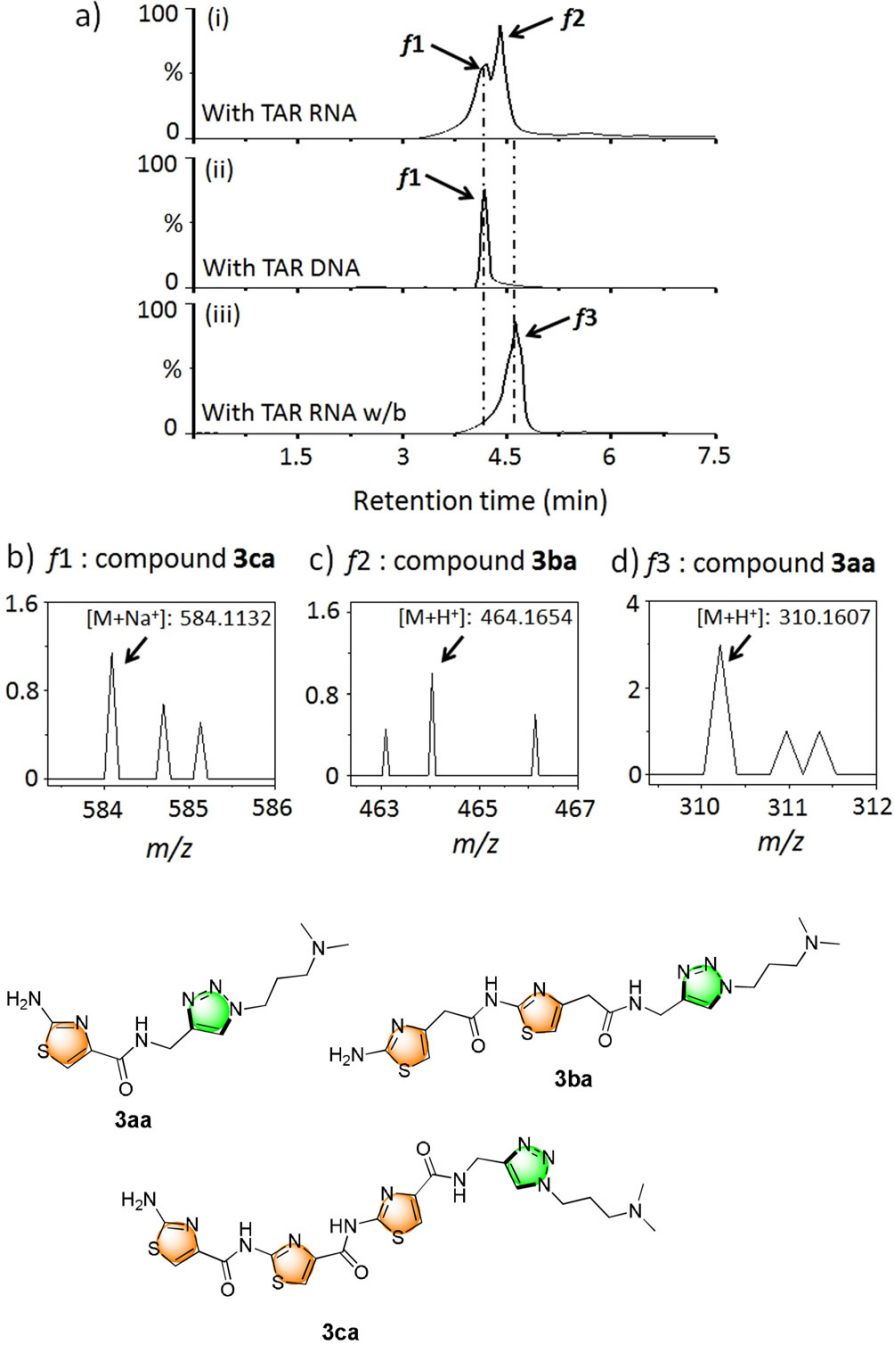
a) In situ formation of hit triazole products analyzed by HPLC using biotin‐tagged i) TAR RNA, ii) TAR DNA, and iii) TAR RNA w/b. ESI‐MS analysis of the fractions collected from the HPLC analysis reveals the formation of compounds b) **3 ca** (*f*1 of TAR RNA) c) **3 ba** (*f*2 of TAR RNA), and d) **3 aa** (*f*3 of TAR RNA w/b), along with their respective molecular structures.

Hit triazole products **3 aa**, **3 ba**, and **3 ca** were prepared from their respective alkyne and azide fragments under Cu^I^‐catalyzed conditions (Figure S2b).^[18]^ The products were obtained in high yields as *anti*‐(1,4‐triazole) regioisomers. Comparison of the HPLC traces of the RNA‐templated products with the authentic 1,4‐triazole samples (Cu^I^‐catalyzed products) revealed that the in situ reaction generates predominantly the *anti*‐(1,4‐triazole) isomers.^[19]^

The yields of the lead *anti*‐triazoles were further determined by time‐dependent cycloaddition of the corresponding alkyne (**1 a**, **1 b**, and **1 c**) and azide (**2 a**) fragments in the presence of the biotin‐tagged target and control DNA and RNA targets (Figure S4). Alkyne **1 b** reacted with azide **2 a** in the presence of TAR RNA to form *anti*‐**3 ba** in 62 % yield (Figure S3a). Similarly, lead *anti*‐**3 ca** (from **1 c** and **2 a**) was obtained in 54 % yield with TAR RNA (Figure S3b). However, in the presence of TAR DNA, *anti*‐**3 ca** was formed in 42 % yield. Lead *anti*‐**3 aa** (from **1 a** and **2 a**) was formed in 51 % yield in the presence of the control TAR RNA w/b.

Analysis of the binding properties of the hit triazole products against the target TAR RNA and controls were performed using isothermal titration calorimetry (ITC) studies (Figure 5 a,b, see also Figure S5). Ligand **3 ba** showed an exothermic binding interaction with TAR RNA with a dissociation constant (*K*_d_) of 0.49 μm. In comparison, ligand **3 ca** showed an approximately 15‐fold lower binding affinity, with *K*_d_=7.2 μm for the TAR RNA (Table 1). Both ligands were found to bind to TAR RNA with a 1:1 stoichiometry (N) and the Δ*G* values were found to be −9.54 kcal mol^−1^ for **3 ba** and −7.01 kcal mol^−1^ for **3 ca**. Moreover, **3 ba** interacted neither with the TAR RNA w/b nor with the TAR DNA. However, **3 ca** exhibited exothermic binding interactions with TAR DNA, with *K*_d_= 11.1 μm and Δ*G*=−6.76 kcal mol^−1^. Ligand **3 aa** showed exothermic binding interactions with TAR RNA w/b, with *K*_d_=9.4 μm and Δ*G*=−6.23 kcal mol^−1^ and didn't interact with the TAR RNA or the TAR DNA. These results indicate that, in contrast to the other triazole leads **3 aa** and **3 ca**, the lead compound **3 ba** preferentially binds to the target TAR RNA.

**Figure 5:**
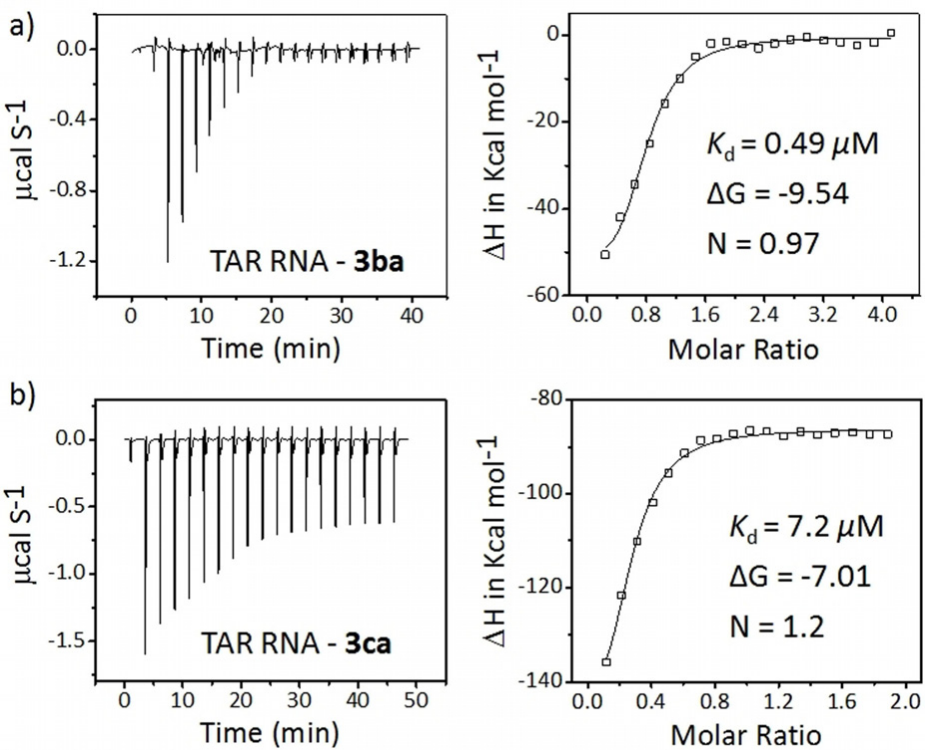
Heat‐burst curves and binding isotherm profiles obtained from ITC studies of ligands a) **3 ba** and b) **3 ca** with TAR RNA performed in the presence of 20 mm Na cacodylate, 180 mm NaCl, and 10 mm MgCl_2_ buffer pH 7.4.

**Table 1:** Binding interactions of triazole leads (**3 aa**, **3 ba**, and **3 ca**) with TAR RNA, TAR DNA, and TAR RNA w/b.

RNA and DNA	Hit ligands	*K*_d_ [μm]^[a]^	Δ*G* [kcal mol^−1^]	*F*/*F*_0_	*K*_d_ [μm]^[b]^	DC_50_ [μm]
TAR RNA	**3 aa**	–	–	1.6	–	–
	**3 ba**	0.49	−9.54	5.0	0.54	1.5
	**3 ca**	7.2	−7.01	5.2	6.7	10.6
						
TAR DNA	**3 aa**	–	–	1.7	–	–
	**3 ba**	–	–	1.5	–	–
	**3 ca**	11.1	−6.76	4.3	10.7	–
						
TAR RNA w/b	**3 aa**	9.4	−6.23	3.8	11.3	–
	**3 ba**	–	–	1.8	–	–
	**3 ca**	–	–	1.7	–	–

[a,b] ITC and fluorescence experiments were conducted in 20 mm sodium cacodylate, 180 mm NaCl, and 10 mm MgCl_2_ at pH 7.4. **3 aa**: *λ*_ex_=280 nm, *λ*_em_=435 nm; **3 ba**: *λ*_ex_=280 nm, *λ*_em_=393 nm, and 410 nm; **3 ca**: *λ*_ex_=280 nm, *λ*_em_=435 nm. *K*_d_ values are determined from [a] ITC and [b] fluorescence titrations (*K*_d_ *=*±5 %).

The binding specificity of the hit triazoles was further analyzed using a fluorescence titration assay. Upon excitation at *λ*=280 nm, **3 ba** showed weak emission maxima at *λ*=393 and 410 nm, while **3 aa** and **3 ca** showed maxima at *λ*=435 nm (Figure S6). Ligands **3 ba** and **3 ca** show an increase in their fluorescence intensity of approximately 5.1‐ and 5.3‐fold respectively, upon titration with TAR RNA (Figure 6 a). However, ligand **3 ba** showed an improved binding with *K*_d_=0.54 μm compared to **3 ca** with *K*_d_=6.7 μm (Table 1). Moreover, ligand **3 ca** also displayed considerable binding interactions with the control TAR DNA and showed a 4.3‐fold increase in fluorescence intensity and *K*_d_=10.74 μm (Figure S6a). In comparison, **3 ba** didn't result in a significant increase in the fluorescence intensity in the presence of TAR DNA and TAR RNA w/b (Table 1), which is in agreement with the ITC results. In addition, **3 aa** exhibited a 3.8‐fold increase in fluorescence intensity with TAR RNA w/b with *K*_d_= 11.3 μm, and didn't interact with the TAR RNA or the control TAR DNA (Figure S6b).

**Figure 6:**
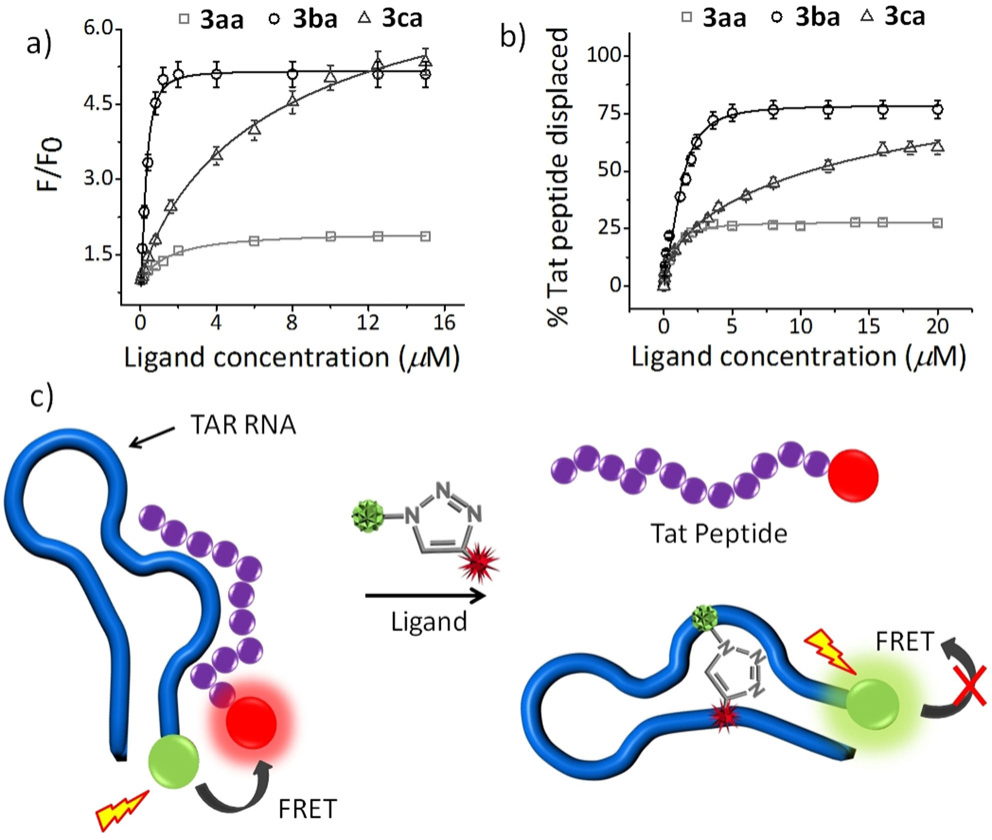
a) Fluorescence titration of hit triazole ligands **3 aa**, **3 ba**, and **3 ca** (2 μm) with TAR RNA (0–8 equiv). b) Percentage of Tat peptide (500 nm) displaced from TAR RNA (500 nm) upon titration with the hit triazole ligands **3 aa**, **3 ba**, and **3 ca** (0–40 equiv). The fluorescence titration and displacement studies were performed with 20 mm Na cacodylate, 180 mm NaCl, and 10 mm MgCl_2_ buffer pH 7.4. c) Schematic representation of the FRET‐based TAR‐Tat peptide displacement assay.

We further assessed the ability of the lead *anti*‐triazole ligands to displace the Tat peptide from a preformed complex between fluoresceinated carboxytetramethylrhodamine (TAMRA) labeled Tat peptide and fluorescein amidite (FAM) labeled TAR RNA by a Förster resonance energy transfer (FRET) based assay (Figure 6 c).^[20]^ In the absence of ligands, the association of Tat with TAR RNA results in an efficient quenching of the FAM dye. The addition of the ligand may displace the Tat peptide, thereby resulting in an increase in the fluorescence emission of the FAM dye (Figure 6 c, see also Figure S7). The displacement of Tat from TAR is expressed as DC_50_, which reflects a ligand's ability to displace 50 % of the bound Tat peptide from the TAR RNA. Ligand **3 ba** showed a lower DC_50_ value (1.5 μm; Figure 6 b) than ligand **3 ca** (DC_50_=10.6 μm), which indicates **3 ba** has a higher affinity for TAR RNA. However, ligand **3 aa** wasn't able to displace 50 % of the bound Tat peptide from the TAR RNA (Table 1), which suggests that the ligand **3 ba** may displace the peptide by binding to the UCU bulge of TAR RNA. The biophysical experiments—ITC, fluorescence, and displacement studies—are thus in agreement with the in situ cycloaddition results.

Molecular docking of **3 ba** and **3 ca** with the target RNA (PDB: 1ANR) was performed with the AutoDock program v4.1 to elucidate the structure–activity relationship of the ligands with the RNA structure (Figure 7, see also Figure S8). The modeling study revealed that *anti*‐**3 ba** could bind to the bulge region of the target while *anti*‐**3 ca** interacted with the major groove region of the target RNA. The presence of a ‐CH_2_ linker provided added flexibility to the first thiazole ring of *anti*‐**3 ba**, thereby making it favorable for interacting with the bulge region of the TAR RNA. The water‐soluble chain of *anti*‐**3 ba** interacted with the phosphate backbone of the other strand, thus making the interaction even more stable (Δ*G*=−9.12 kcal mol^−1^). *Anti*‐**3 ca**, on the other hand, exhibited a planar crescent‐shaped structure which stacks between the major groove of the TAR RNA (Δ*G*=−6.85 kcal mol^−1^) and TAR DNA. The bulge region further widens the groove (in TAR RNA and DNA) and makes the stacking interactions more favorable but nonspecific for *anti*‐**3 ca**.

**Figure 7:**
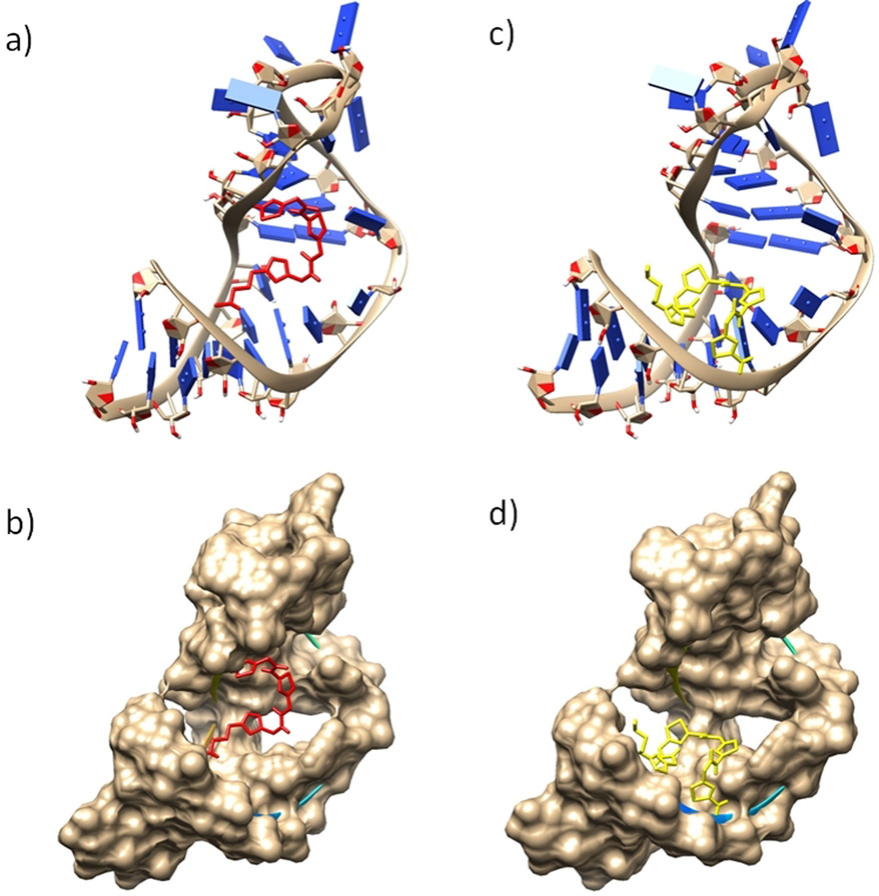
Molecular docking of ligands **3 ba** (a,b) and **3 ca** (c,d) with TAR RNA (PDB ID: 1ANR) using the AutoDock 4.0 program; the side view of RNA is shown.

In summary, in situ click chemistry has been successfully applied to discover novel thiazole‐based peptidomimetics as HIV‐1 TAR‐Tat inhibitors. The TAR RNA itself acts as a template for the cycloaddition and promotes the formation of a thiazole peptidomimetic 1,4‐triazole lead that shows high selectivity for TAR RNA. Isothermal calorimetric and fluorescence studies reveal that a thiazole peptidomimetic with two thiazole rings and flexible methylene units strongly interacts with the bulge region of TAR RNA and effectively inhibits the Tat‐TAR RNA interactions. Further studies are currently underway to apply this method for the discovery of novel TAR RNA inhibitors and to investigate their antiviral activities.

## Conflict of interest

The authors declare no conflict of interest.

## Supporting information

As a service to our authors and readers, this journal provides supporting information supplied by the authors. Such materials are peer reviewed and may be re‐organized for online delivery, but are not copy‐edited or typeset. Technical support issues arising from supporting information (other than missing files) should be addressed to the authors.

Supplementary
