# Supplementary material for: Target‐Directed Azide‐Alkyne Cycloaddition for Assembling HIV‐1 TAR RNA Binding Ligands
Source: Angew Chem Int Ed Engl. 2020 May 25;59(30):12407–11. doi: 10.1002/anie.202003461 (PMC7687225; doi:10.1002/anie.202003461)
Supplement: Supplementary file 1 — Supplementary [file ANIE-59-12407-s001.pdf]

Angewandte Chemie
Eine Zeitschrift der Gesellschaft Deutscher Chemiker

Supporting Information

# Target-Directed Azide-Alkyne Cycloaddition for Assembling HIV-1 TAR RNA Binding Ligands

*Rakesh Paul, Debasish Dutta, Raj Paul, and Jyotirmayee Dash**

anie_202003461_sm_miscellaneous_information.pdf

**Table of contents**

1.0 General information S2

2.0 Synthesis of thiazole peptides S3

3.0 Synthesis of alkyne and azide fragments S4

4.0 NMR spectra of the fragments S14

5.0 Oligonucleotide sequences used in *in situ* reaction S20

6.0 General procedure for *in situ* cycloaddition using biotin tagged TAR RNA and control TAR RNA w/b & TAR DNA S20

7.0 Monitoring the *in situ* cycloaddition by HPLC and ESI-MS S21

8.0 Relative yields of triazole leads S22

9.0 Synthesis of thiazole peptidomimetics S24

10.0 NMR spectra of all compounds S29

11.0 Isothermal calorimetric titrations S35

12.0 Fluorescence titration studies S36

13.0 Fluorescence displacement studies S38

14.0 Molecular docking S38

## 1.0 General information

All experiments were carried out under an inert atmosphere of argon in flame-dried flasks. Solvents were dried using standard procedures. All starting materials were obtained from commercial suppliers and used as received. Products were purified by flash chromatography on silica gel (100-200 mesh, Merck). Unless otherwise stated, yields refer to analytical pure samples. Melting points were measured with BÜCHI Melting point B-545 and are uncorrected. NMR spectra were recorded in $CDCl_3$ unless otherwise stated. **$^1$H NMR** spectra were recorded at 500 MHz using Brüker AVANCE 500 MHz and JEOL 400 MHz instruments at 298 K. Signals are quoted as δ values in ppm using residual protonated solvent signals as internal standard ($CDCl_3$: δ 7.26 ppm). Data is reported as follows: chemical shift, integration, multiplicity (s = singlet, d = doublet, t = triplet, q = quartet, p = pentet, br = broad, m = multiplet), and coupling constants (Hz). **$^{13}$C NMR** spectra were recorded on either a JEOL-400 (100 MHz), or a Brüker AVANCE 500 MHz (125 MHz) with complete proton decoupling. Chemical shifts (δ) are reported in ppm downfield from tetramethylsilane with the solvent as the internal reference ($CDCl_3$: δ 77.16 ppm). Infrared (FTIR) spectra ($\nu_{max}$) are recorded on a Perkin Elmer spectrophotometer Spectrum RX1 using KBr disk techniques for solid compounds and as a thin film (neat) for liquid samples and are reported in $cm^{-1}$. **HRMS** analyses were performed with Q-TOF YA263 high resolution (Water Corporation) instruments by +ve mode electrospray ionization.

## 2.0 Synthesis of thiazole peptides

The thiazole peptides were synthesized by step-wise amide coupling of thiazole amino acid building blocks **3** and **5**. The thiazole building block **3** was prepared in 90% yield by refluxing thiourea **1** and ethyl bromopyruvate **2** in dry ethanol at 100 °C. The Boc-protection of thiazole compound **3** followed by the ester hydrolysis of the resulting ester **4** yielded the acid building block **5** (Scheme S1). The amide coupling of thiazole amino acid building blocks **3** and **5** were carried out using HBTU in the presence of DIEA in anhydrous $CH_2Cl_2$ to afford the dipeptide **6** in 80% yield. Subsequent ester hydrolysis of **6** followed by an amide coupling of the resulting acid **7** with the building block **3** afforded the tripeptide **8** in 55% yield. Ester hydrolysis of tripeptide **8** using LiOH yielded the trimeric acid **9** (Scheme S1).

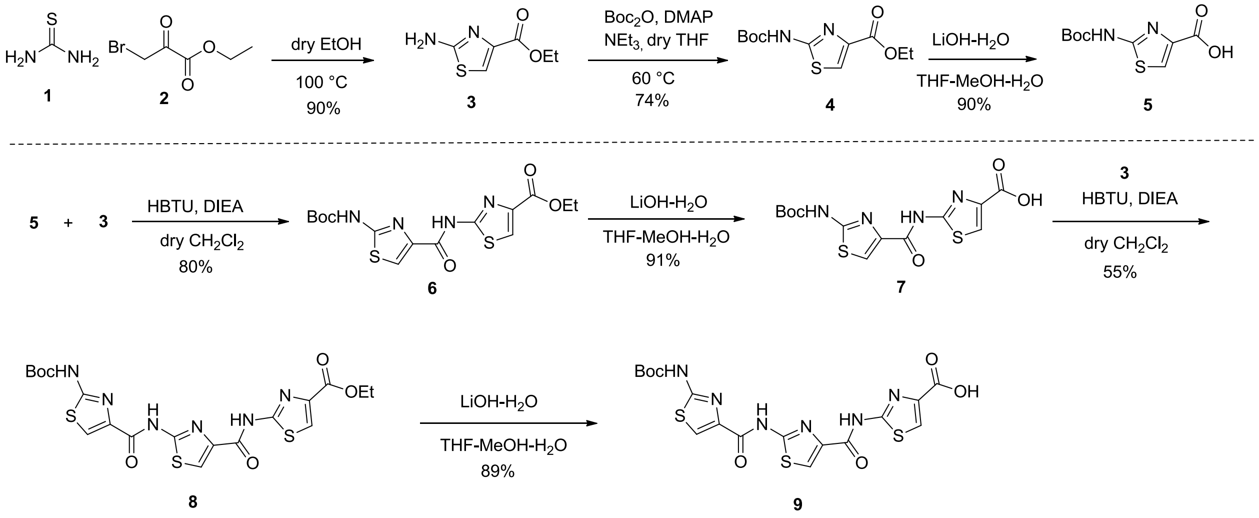


**Scheme S1.** Synthesis of thiazole peptides.

The thiazole building block **12** was prepared in two steps starting from ethylacetoacetate **10**. The bromination of ethyl acetoacetate **10** afforded ethyl γ-bromoacetoacetate **11** in 65% yield. The desired building block (2-aminothiazol-4-yl)acetic acid ethyl ester **12** was prepared in 75% yield by refluxing thiourea **1** and ethyl γ-bromoacetoacetate **11** in dry ethanol at 100 °C. The Boc-protection followed by the ester hydrolysis of the resulting ester **13** yielded the acid building block **14**. The amide coupling of thiazole amino acid building blocks **12** and **14** was carried out using HBTU in the presence of DIEA in anhydrous $CH_2Cl_2$ to afford the dipeptide **15** in 85% yield. Ester hydrolysis of dipeptide **15** using LiOH provided the acid **16** (Scheme S2).

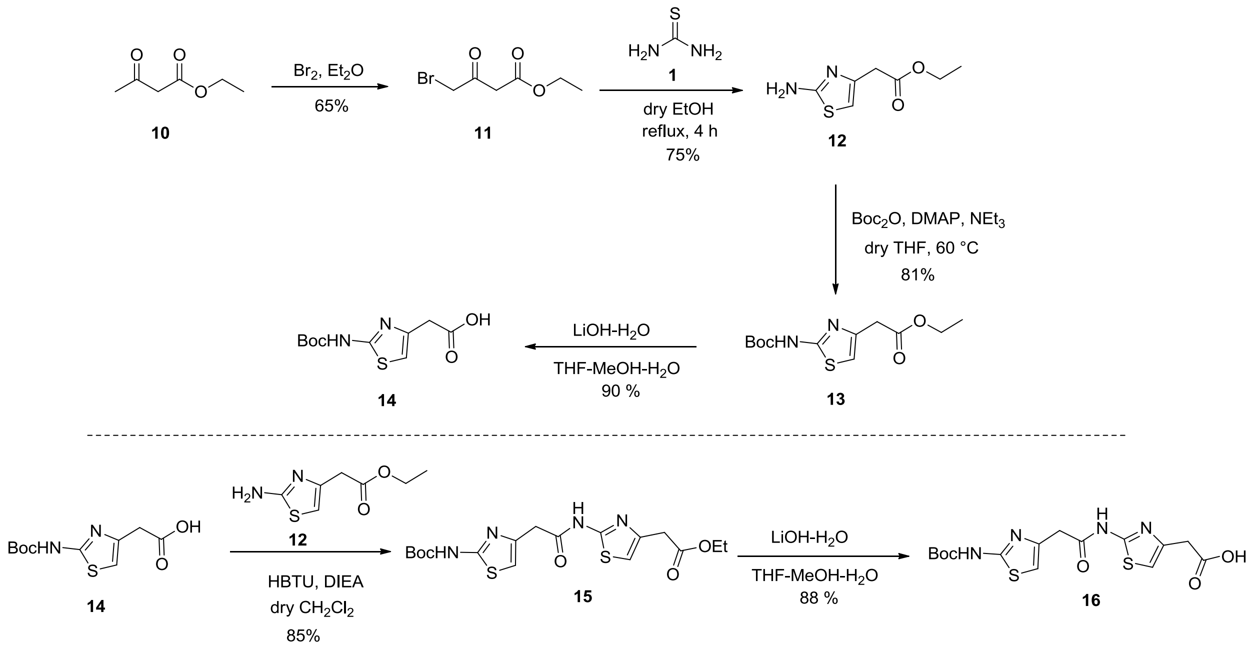


**Scheme S2.** Synthesis of thiazole peptides.

## 3.0 Synthesis of alkyne and azide fragments

**Synthesis of thiazole containing alkyne fragments (1a-c):** Propargyl amine **17** was coupled with three different thiazole acids **5**, **16** and **9** using HBTU in the presence of DIEA in anhydrous $CH_2Cl_2$ to afford the thiazole peptides **18**, **19** and **20** respectively and then the boc-deprotection of the synthesized peptides **18**, **19** and **20** using TFA in $CH_2Cl_2$ afforded the alkyne fragments **1a**, **1b** and **1c** respectively (Scheme S3).

**Synthesis of alkyne 1d:** Synthesis of alkyne **1d** has been reported previously.[1]

[1] D. Panda, P. Saha, T. Das, J. Dash, *Nat. commun.*, **2017**, *8*, 1-11.

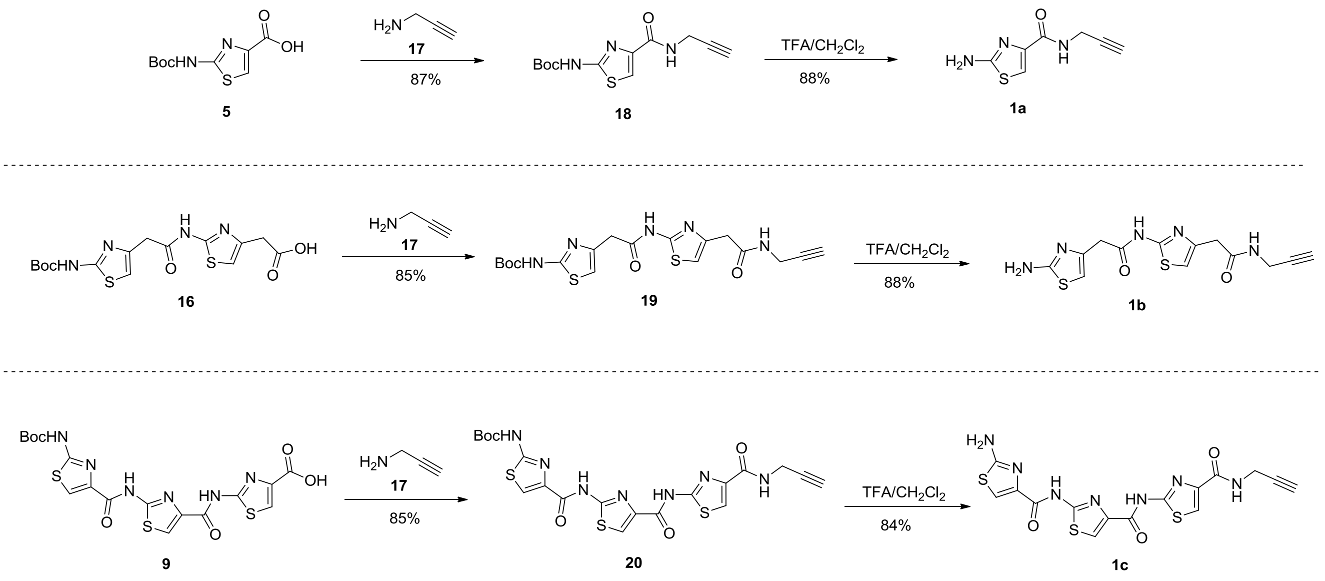

**Scheme S3.** Synthesis of thiazole containing alkyne fragments.

**General procedure for the deprotection of the ethyl ester (GP-1):** To a solution of the respective ethyl ester protected peptide (1 eq) in THF/MeOH/$H_2O$ = 3: 3: 1, LiOH-$H_2O$ (3 eq) was added at 0 °C. The reaction mixture was stirred for about 3-4 h at room temperature until starting material was fully consumed. Reaction was monitored by TLC. After completion of the reaction, solvent was dried in rotary evaporator. The crude obtained was dissolved in little amount of water followed by drop wise addition of saturated $KHSO_4$ solution under cold conditions that allowed the reaction mixture to get precipitated under acidic conditions. The resulting solid precipitate was filtered and dried to afford the corresponding hydrolysed compounds as white solids in quantitative yield.

**General procedure for the peptide coupling (GP-2):** All amide coupling reactions were carried out using O-(Benzotriazol-1-yl)-*N*, *N*, *N′*, *N′*-tetramethyluronium hexafluorophosphate (HBTU) as the peptide coupling reagent as it provided the desired coupled products in high yields. To a stirred solution of carboxylic acid component (1.1 eq) in dry $CH_2Cl_2$, HBTU (1.5 eq) was added followed by the addition of *N*, *N′*-diisopropylethylamine (DIEA) (3 eq) at 0 ºC. After 10 minutes, the amine component (1 eq) was added at same temperature. The reactions were typically allowed to stir for 16-24 h at room temperature. After completion of the reaction, the reaction mixture was concentrated and the resulting residue was dissolved in ethylacetate and the

organic layer was successively washed thrice with 1(N) HCl solution, saturated $NaHCO_3$ solution and brine. After drying with $Na_2SO_4$, the solvents were removed under reduced pressure and the desired coupling products were purified by column chromatography. Yields for the individual coupling steps were good to excellent ranging from 55% to 85%.

**General procedure for the peptide coupling (GP-3):** To a stirred solution of carboxylic acid component (1 eq) in dry $CH_2Cl_2$, HBTU (1.5 eq) was added followed by the addition of *N*, *N′*-diisopropylethylamine (DIEA) (3 eq) at 0 ºC. After 10 minutes, the amine component (1.1 eq) was added at same temperature. The reactions were typically allowed to stir for 24 h at room temperature. After completion of the reaction, the reaction mixture was diluted with $CH_2Cl_2$. The organic layer was successively washed with saturated $NaHCO_3$ solution (3× 20 mL) and brine. After drying with $Na_2SO_4$, the solvents were removed under reduced pressure and the desired coupling products were purified by column chromatography in excellent yield.

**General procedure for the deprotection of the Boc-group (GP-4):** The respective NH-Boc protected thiazole peptides were dissolved in $CH_2Cl_2$ and cooled to 0 °C. TFA (equal amount as the solvent) was added and the solution was allowed to warm to room temperature. The reaction mixture was stirred for about 3-4 h at room temperature until starting material was fully consumed. Reaction was monitored by TLC. After completion of the reaction, the reaction mixture was neutralised with aqueous ammonia solution and Boc-deprotected thiazole peptides were purified by column chromatography to provide the corresponding products in quantitative yields.

**General procedure for the synthesis of triazole derivatives using click chemistry (GP-5):** Alkyne (1 eq) was dissolved in a 3:1 mixture of *t*BuOH/$H_2O$ (3 mL). Copper (II) sulphate pentahydrate (0.1 eq) and sodium ascorbate (0.2 eq) were added and the solution was stirred for 10 min. The corresponding azide (10 eq) was added and the mixture was then allowed to stir for overnight. After completion of the reaction, the reaction mixture was concentrated. The crude product was purified by column chromatography to give the corresponding triazole derivatives.

**Synthesis of ethyl 2-amino-4-thiazolecarboxylate 3:**[2] Ethyl bromopyruvate **2** (5.31 g, 26.3

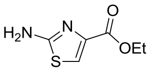

mmol) was added to a cold solution of thiourea **1** (2 g, 26.3 mmol) in dry ethanol (5 mL) in a sealed tube. The resulting mixture was heated for 4 h at 100 °C. Upon cooling to room temperature, the reaction mixture was poured into ice water and brought to pH ~ 8 with aqueous sodium carbonate solution. The resulting solid precipitate was filtered, washed several times with water and air dried to obtain pure yellowish solid compound **3** (4.07 g) in 90% yield. $^1$H NMR (400 MHz, DMSO-d$_6$): 7.45 (1H, s), 7.22 (2H, s$_{br}$), 4.19 (2H, q, $J$ = 7.3 Hz), 1.25 (3H, t, $J$ = 6.7 Hz); $^{13}$C NMR (100 MHz, DMSO-d$_6$): 168.2, 161.0, 142.2, 116.9, 60.1, 14.1; HRMS (ESI) calcd for $C_6H_9N_2O_2S$ [M+H]$^+$: 173.0379; Found: 173.0391.

**Synthesis of Boc-protected thiazole amino ester 4:**[3] To a stirred solution of aminothiazole

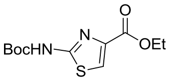

ester **3** (2 g, 11.6 mmol) in tetrahydrofuran (40 mL) at 25 °C was added triethylamine (2.12 mL, 15.2 mmol), 4-(dimethylamino)pyridine (140 mg, 1.16 mmol), and di-*tert*-butyl-dicarbonate (3 mL, 12.7 mmol) sequentially, and the reaction mixture was heated to 60 °C. After 1 h, the reaction mixture was allowed to cool to 25 °C, and quenched with saturated aqueous ammonium chloride solution (50 mL). The two phases were separated, and the aqueous layer was extracted with ethyl acetate (3 × 20 mL). The combined organic layers were dried with Na$_2$SO4 and concentrated under reduced pressure. The obtained residue was purified by column chromatography to afford pure thiazolyl carbamate **4** (2.34 g, 74%) as a white solid. $^1$H NMR (400 MHz, DMSO-d$_6$): 11.77 (1H, s$_{br}$), 7.99 (1H, s), 4.25 (2H, q, $J$ = 6.7 Hz), 1.47 (9H, s), 1.27 (3H, t, $J$ = 7.3 Hz); $^{13}$C NMR (100 MHz, DMSO-d$_6$): 160.9, 159.8, 153.0, 141.3, 122.2, 60.4, 27.8, 14.1; HRMS (ESI) calcd for $C_{11}H_{16}N_2O_4SNa$ [M+Na]$^+$: 295.0728; Found: 295.0728.

**Synthesis of Boc-protected thiazole amino acid 5:** Using general procedure GP-1, LiOH-H$_2$O

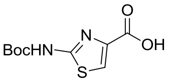

(5.7 g, 136 mmol) and Boc-protected thiazole ester 4 (12.64 g, 45.3 mmol) in THF/MeOH/H$_2$O (60 mL) with stirring for 4 h, provided the corresponding thiazole acid 5 (10.2 g, 90%) as a white solid. $^1$H NMR (400 MHz, DMSO-d$_6$): 7.87 (1H, s), 1.45

[2] R. P. Karuvalam, K. R. Haridas, S. K. Nayak, T. N. G. Row, P. Rajeesh, R. Rishikesan, N. S. Kumari, *Eur. J. Med. Chem*. **2012**, *49*, 172-182.

[3] K. C. Nicolaou, D. Rhoades, Y. Wang, R. Bai, E. Hamel, M. Aujay, J. Sandoval, J. Gavrilyuk, *J. Am. Chem. Soc*. **2017**, *139*, 7318−7334.

(9H, s); $^{13}$C NMR (100 MHz, DMSO-d$_6$): 162.7, 159.8, 153.3, 142.8, 121.9, 81.9, 28.1; HRMS (ESI) calcd for $C_9H_{12}N_2O_4SNa$ [M+Na]$^+$: 267.0415; Found: 267.0403.

**Synthesis of Boc-protected thiazole dipeptide 6**:[3] Using general procedure **GP-2**, Boc protected thiazole amino acid **5** (6.24 g, 25.5 mmol), HBTU (13.19 g, 34.8 mmol), DIEA (12.12 mL, 69.6 mmol) and thiazole amine **3** (4 g, 23.2 mol) in dry $CH_2Cl_2$ (60 mL), with stirring for 16 h provided the desired dipeptide **6** (7.4 g, 80%) as an off-white solid; $^1$H NMR (500 MHz, $CDCl_3$): 10.68 (1H, $s_{br}$), 9.01 (1H, $s_{br}$), 7.85 (1H, s), 7.82 (1H, s), 4.27 (2H, q, $J$ = 7.3 Hz), 1.47 (9H, s), 1.33 (3H, t, $J$ = 6.7 Hz); $^{13}$C NMR (125 MHz, $CDCl_3$): 161.5, 160.2, 159.2, 157.8, 152.3, 142.5, 142.0, 122.5, 120.5, 83.3, 61.4, 28.2, 14.3; HRMS (ESI) calcd for $C_{15}H_{19}N_4O_5S_2$ [M+H]$^+$: 399.0791; Found: 399.0778.

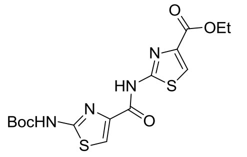


**Synthesis of Boc-protected thiazole dimeric acid 7:** Using general procedure **GP-1**, LiOH-$H_2O$ (2.34 g, 55.7 mmol) and dipeptide **6 (**7.4 g, 18.6 mmol) in THF/MeOH/$H_2O$ (40 mL) with stirring for 4 h, provided the corresponding thiazole acid **7** (6.26 g, 91%) as a white solid; $^1$H NMR (500 MHz, DMSO-d$_6$): 12.29 (1H, $s_{br}$), 11.82 (1H, $s_{br}$), 8.20 (1H, s), 8.03 (1H, s), 1.49 (9H, s); $^{13}$C NMR (100 MHz, DMSO-d$_6$): 162.4, 160.1, 159.4, 157.7, 142.4, 142.3, 122.9, 120.5, 81.9, 27.9; HRMS (ESI) calcd for $C_{13}H_{14}N_4O_5S_2Na$ [M+Na]$^+$: 393.0303; Found: 393.0289.

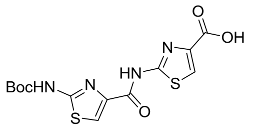


**Synthesis of Boc-protected thiazole tripeptide 8**:[4] Using general procedure **GP-2**, Boc protected thiazole dimeric acid **7** (4.73 g, 12.8 mmol), HBTU (6.6 g, 17.4 mmol), DIEA (6.1 mL, 34.8 mmol) and thiazole amine **3** (2 g, 11.6 mmol) in dry $CH_2Cl_2$ (50 mL), with stirring for 24 h provided the desired tripeptide **8** (3.34 g, 55%) as an off-white solid; $^1$H NMR (500 MHz, DMSO-d$_6$): 8.28 (1H, s), 8.13 (1H, s), 8.08 (1H, s), 4.27 (2H, q, $J$ = 6.9 Hz), 1.49 (9H, s), 1.29 (3H, t, $J$ = 6.9 Hz). $^{13}$C NMR (125 MHz, DMSO-d$_6$): 161.0, 160.0, 159.4, 158.2, 158.0, 156.8, 152.9, 142.4, 142.2, 141.2, 123.3, 121.6, 120.6, 81.9, 60.7, 27.9, 14.2; HRMS (ESI) calcd for $C_{19}H_{20}N_6O_6S_3Na$ [M+Na]$^+$: 547.0504; Found: 547.0469.

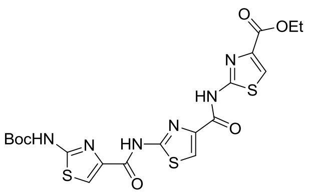


[4] F. Brucoli, R. M. Hawkins, C. H. James, P. J. M. Jackson, G. Wells, T. C. Jenkins, T. Ellis, M. Kotecha, D. Hochhauser, J. A. Hartley, P. W. Howard, *J. Med. Chem.* **2013**, *56*, 6339-6351.

**Synthesis of Boc-protected thiazole trimeric acid 9:** Using general procedure **GP-1**, LiOH-$H_2O$ (0.6 g, 14.2 mmol) and Boc-protected thiazole tripeptide **8** (2.5 g, 4.7 mmol) in THF/MeOH/$H_2O$ (20 mL) with stirring for 4 h, provided the corresponding thiazole acid **9** (2.1 g, 89%) as a white solid; $^1$H NMR (500 MHz, DMSO-d$_6$): 12.65 (1H, s$_{br}$), 12.37 (1H, s$_{br}$), 11.77 (1H, s$_{br}$), 8.32 (1H, s), 8.19 (1H, s), 8.05 (1H, s), 1.51 (9H, s); $^{13}$C NMR (100 MHz, DMSO-d$_6$): 162.3, 160.1, 159.4, 159.3, 158.0, 157.7, 153.0, 142.9, 142.3, 142.2, 122.9, 121.5, 120.7, 81.9, 27.8; HRMS (ESI) calcd for $C_{17}H_{16}N_6O_6S_3Na$ [M+Na]$^+$: 519.0191; Found: 519.0191.

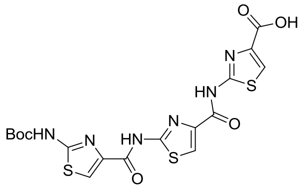


**Synthesis of ethyl 4-bromo-3-oxobutanoate 11.** To a solution of ethyl acetoacetate **10** (30 g, 230 mmol) in dry $Et_2O$ (50 mL) at 0 °C was added bromine (12 mL, 230 mmol) drop wise over 45 min with vigorous stirring. After the mixture was stirred at room temperature for 24 h, ice was added, and the organic phase washed with sodium hydrogen carbonate solution and twice with saturated sodium chloride solution (2 × 200 mL). The organic phase was dried over $Na_2SO_4$ for 2 h, and the solvent was removed under reduced pressure to give orange oil (31.3 g, 65%), which was immediately used for the next step without any purification.

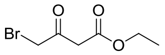


**Synthesis of ethyl 2-aminothiazole-4-acetate 12.** Ethyl 4-bromo-3-oxobutanoate **11** (31.3 g, 150 mmol) was dissolved in dry ethanol (100 mL), the reaction mixture was cooled and thiourea **1** (11.4 g, 150 mmol) was added to it. After 10 minutes, the reaction mixture was allowed to warm to room temperature and the reaction mixture was refluxed for 5 h at 100 $^\circ$C. The mixture was cooled and concentrated under reduced pressure. After neutralization with aq. $NaHCO_3$, the aqueous layer was extracted with EtOAc (3 x 150 mL). The combined organic layers were dried over $Na_2SO_4$ and evaporated in vacuo. The crude material was purified by column chromatography to afford **12** (20.9 g, 75%) as a yellow solid. $^1$H NMR (400 MHz, $CDCl_3$): 6.32 (1H, s), 5.30 (1H, s$_{br}$), 4.17 (2H, q, $J$ = 7.3 Hz), 3.54 (2H, s), 1.26 (3H, t, $J$ = 7.3 Hz); $^{13}$C NMR (100 MHz, $CDCl_3$): 170.6, 167.9, 144.7, 105.5, 61.1, 37.4, 14.3; HRMS (ESI) calcd for $C_7H_{10}N_2O_2S$ [M+H]$^+$: 187.0535; Found: 187.0534.

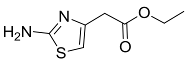

**Synthesis of Boc-protected thiazole amino ester 13:** To a stirred solution of amino thiazole

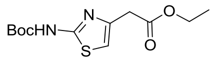

ester **12** (10 g, 53.7 mmol) in tetrahydrofuran (80 mL) at 25 °C was added triethylamine (9.8 mL, 69.8 mmol), 4-(dimethylamino)pyridine (656 mg, 5.37 mmol), and di-*tert*-butyl-dicarbonate (13.5 mL, 59.07 mmol) sequentially, and the reaction mixture was heated to 60 °C. After 1 h, the reaction mixture was allowed to cool to 25 °C, and quenched with saturated aqueous ammonium chloride solution (50 mL). The two phases were separated, and the aqueous layer was extracted with ethyl acetate (3 × 50 mL). The combined organic layers were dried with $Na_2SO_4$ and concentrated under reduced pressure. The obtained residue was purified by column chromatography to afford pure thiazolyl carbamate **13** (12.4 g, 81%) as a white solid. $^1H$ NMR (500 MHz, $CDCl_3$): 10.89 (1H, $s_{br}$), 6.74 (1H, s), 4.12 (2H, q, $J$ = 6.9 Hz), 3.76 (2H, s), 1.52 (9H, s), 1.18 (3H, t, $J$ = 6.9 Hz); $^{13}C$ NMR (125 MHz, $CDCl_3$): 170.2, 161.1, 152.6, 143.5, 109.3, 82.6, 60.8, 36.9, 28.3, 14.1; HRMS (ESI) calcd for $C_{12}H_{18}N_2O_4S$ $[M+H]^+$: 287.1060; Found: 287.1063.

**Synthesis of Boc-protected thiazole amino acid 14**: Using general procedure **GP-1**, LiOH-$H_2O$

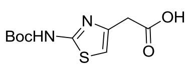

(5.3 g, 126 mmol) and Boc-protected thiazole ester **13 (**12 g, 41.9 mmol) in THF/MeOH/$H_2O$ (40 mL) with stirring for 4 h, provided the corresponding thiazole acid **14** (8.1 g, 90%) as a white solid. $^1H$ NMR (500 MHz, $CDCl_3$): 6.69 (1H, s), 3.65 (2H, s), 1.54 (9H, s); $^{13}C$ NMR (100 MHz, $CDCl_3$): 174.7, 162.3, 152.6, 142.3, 109.9, 82.9, 36.8, 28.3; HRMS (ESI) calcd for $C_{10}H_{14}N_2O_4SNa$ $[M+Na]^+$: 281.0572; Found: 281.0566.

**Synthesis of Boc-protected thiazole dipeptide 15**: Using general procedure **GP-2**, Boc

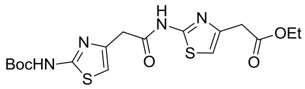

protected thiazole amino acid **14** (6.1 g, 23.65 mmol), HBTU (12.22 g, 32.5 mmol), DIEA (11.22 mL, 64.4 mmol) and thiazole amine **12** (4 g, 21.5 mmol) in dry $CH_2Cl_2$ (60 mL) with stirring for 16 h provided the desired dipeptide **15** (7.78 g, 85%) as an off-white solid; $^1H$ NMR (500 MHz, $CDCl_3$): 11.57 (1H, $s_{br}$), 10.34 (1H, $s_{br}$), 6.77 (1H, s), 6.76 (1H, s), 4.12 (2H, q, $J$ = 7.5 Hz), 3.81 (2H, s), 3.67 (2H, s), 1.55 (9H, s), 1.20 (3H, t, $J$ = 6.9 Hz); $^{13}C$ NMR (100 MHz, $CDCl_3$): 170.3, 167.2, 161.9, 158.4, 152.5, 143.2, 142.4, 110.8, 83.0, 61.2, 38.5, 37.1, 28.3, 14.2; HRMS (ESI) calcd for $C_{17}H_{22}N_4O_5S_2Na$ $[M+Na]^+$: 449.0929; Found: 449.0926.

**Synthesis of Boc-protected thiazole dimeric acid 16:** Using general procedure **GP-1**, LiOH-$H_2O$ (1.8 g, 42.2 mmol) and dipeptide **15** (6.0 g, 14.06 mmol) in THF/MeOH/$H_2O$ (30 mL) with stirring for 4 h, provided the corresponding thiazole acid **16** (4.93 g, 88%) as a white solid; $^1$H NMR (500 MHz, DMSO-d$_6$): 12.26 (1H, s$_{br}$), 11.39 (1H, s$_{br}$), 6.93 (1H, s), 6.92 (1H, s), 3.74 (2H, s), 3.59 (2H, s), 1.46 (9H, s); $^{13}$C NMR (100MHz, DMSO-d$_6$): 171.6, 167.9, 159.5, 157.4, 152.8, 144.3, 144.2, 110.1, 109.8, 81.0, 37.8, 36.8, 27.9; HRMS (ESI) calcd for $C_{15}H_{18}N_4O_5S_2Na$ [M+Na]$^+$: 421.0616; Found: 421.0615.

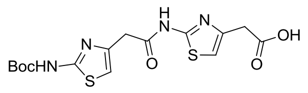


**Synthesis of alkyne containing thiazole peptide 18:** Using general procedure **GP-3**, Boc protected thiazole acid **5** (300 mg, 1.23 mmol), HBTU (698 mg, 1.84 mmol), DIEA (0.642 mL, 3.69 mmol) and propargylamine **17** (95 µL, 1.476 mmol) in dry $CH_2Cl_2$ (15 mL) provided the desired compound **18** (300.6 mg, 87 %) as a colorless solid; $^1$H NMR (300 MHz, DMSO-d$_6$): 11.64 (s, 1H), 8.10 (t, *J* = 5.9 Hz, 1H), 7.77 (s, 1H), 4.03 (dd, *J* = 5.9, 2.5 Hz, 2H), 3.11 (t, *J* = 2.4 Hz, 1H), 1.49 (s, 9H); $^{13}$C NMR (75 MHz, DMSO-d$_6$): 160.4, 159.7, 153.0, 144.3, 117.3, 81.6, 81.2, 72.8, 27.8; HRMS (ESI) calcd for $C_{12}H_{15}N_3O_3SNa$ [M+Na]$^+$: 304.0732; Found: 304.0734.

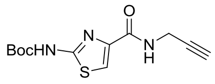


**Synthesis of alkynated thiazole peptide 19:** Using general procedure **GP-3**, Boc protected thiazole acid **16** (400 mg, 1.003 mmol), HBTU (570 mg, 1.504 mmol), DIEA (0.52 mL, 3.01 mmol) and propargylamine **17** (71 µL, 1.103 mmol) in dry $CH_2Cl_2$ (15 mL) provided the desired compound **19** (372 mg, 85%) as an off-white solid; $^1$H NMR (400 MHz, DMSO-d$_6$): 12.27 (1H, s$_{br}$), 11.42 (1H, s$_{br}$), 8.39 (1H, t, *J* = 4.9 Hz), 6.92 (1H, s), 6.72 (1H, s), 6.88 (1H, s), 3.87-3.85 (2H, m), 3.70 (2H, s), 3.48 (2H, s), 3.11 (1H, t, *J* = 2.4 Hz), 1.46 (9H, s); $^{13}$C NMR (125 MHz, DMSO-d$_6$): 168.6, 167.8, 159.4, 157.3, 152.7, 144.9, 144.2, 109.7, 109.6, 81.0, 80.9, 72.8, 37.9, 37.8, 27.9, 27.8; HRMS (ESI) calcd for $C_{18}H_{21}N_5O_4S_2Na$ [M+Na]$^+$: 458.0933; Found: 458.0935.

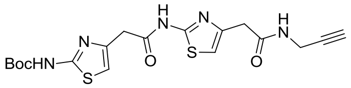


**Synthesis of alkyne containing thiazole peptide 20:** Using general procedure **GP-3**, Boc protected thiazole acid **9** (300 mg, 0.6 mmol), HBTU (341 mg, 0.9 mmol), DIEA (0.316 mL, 1.8 mmol) and propargylamine **17** (46 µL, 0.72 mmol) in dry $CH_2Cl_2$ (15 mL) provided the desired

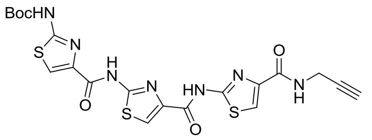

compound **20** (274 mg, 85 %) as a white solid; $^1$H NMR (500 MHz, DMSO-d$_6$): 12.32 (1H, s$_{br}$), 12.08 (1H, s$_{br}$), 11.78 (1H, s$_{br}$), 8.39 (1H, t, *J* = 5.7 Hz), 8.30 (1H, s), 8.22 (1H, s), 7.91 (1H, s), 4.07 (2H, dd, *J* = 2.5, 3.1 Hz), 3.13 (1H, s), 1.51 (9H, s); $^{13}$C NMR (100 MHz, DMSO-d$_6$): 160.3, 160.1, 159.3, 159.1, 157.9, 157.4, 152.9, 144.2, 142.2, 142.1, 121.5, 120.7, 118.4, 81.8, 81.0, 72.9, 28.2, 27.8; HRMS (ESI) calcd for $C_{20}H_{20}N_7O_5S_3$ $[M+H]^+$: 534.0683; Found: 534.0686.

**Synthesis of thiazole containing alkyne 1a:** Using general procedure **GP-4**, trifluoroacetic acid

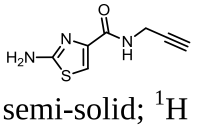


(3 mL) and Boc protected thiazole alkyne **18** (300 mg, 1.066 mmol) in $CH_2Cl_2$ (3 mL) provided the corresponding alkyne **1a** (170 mg, 88%) as a colorless semi-solid; $^1$H NMR (300 MHz, DMSO-*d*$_6$): 8.08 (t, *J* = 5.9 Hz, 1H), 7.22 (s, 1H), 7.08 (s, 2H), 3.96 (dd, *J* = 6.0, 2.5 Hz, 2H), 3.05 (t, *J* = 2.4 Hz, 1H); $^{13}$C NMR (75 MHz, DMSO-*d*$_6$): 168.2, 160.6, 145.2, 111.8, 81.3, 72.5, 28.0; HRMS (ESI) calcd for $C_7H_7N_3OSNa$ $[M+Na]^+$: 204.0208; Found: 204.0206.

**Synthesis of thiazole containing alkyne 1b:** Using general procedure **GP-4**, trifluoroacetic acid

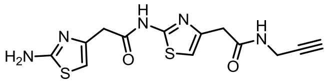

(3 mL) and Boc protected thiazole alkyne **19** (300 mg, 0.689 mmol) in $CH_2Cl_2$ (3 mL) provided the corresponding alkyne **1b** (203 mg, 88%) as a white solid; $^1$H NMR (300 MHz, DMSO-*d*$_6$): 12.17 (s, 1H), 8.38 (t, *J* = 5.5 Hz, 1H), 6.92 (s, 2H), 6.87 (s, 1H), 6.32 (s, 1H), 3.86 (dd, *J* = 5.5, 2.6 Hz, 2H), 3.56 (s, 2H), 3.48 (s, 2H), 3.11 (t, *J* = 2.5 Hz, 1H); $^{13}$C NMR (75 MHz, DMSO-*d*$_6$): 168.7, 168.3, 168.1, 157.4, 145.0, 144.8, 109.8, 103.0, 81.1, 73.0, 38.1, 38.0, 28.0; HRMS (ESI) calcd for $C_{13}H_{13}N_5O_2S_2Na$ $[M+Na]^+$: 358.0408; Found: 358.0406.

**Synthesis of thiazole containing alkyne 1c:** Using general procedure **GP-4**, trifluoroacetic acid

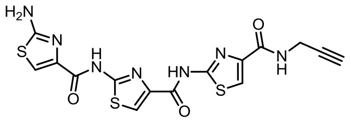

(3 mL) and Boc protected thiazole alkyne **20** (300 mg, 0.562 mmol) in $CH_2Cl_2$ (3 mL) provided the corresponding alkyne **1c** (204 mg, 84%) as a white solid; $^1$H NMR (500 MHz, DMSO-*d*$_6$): 12.01 (s, 1H), 11.78 (s, 1H), 8.38 (t, *J* = 5.8 Hz, 1H), 8.31 (s, 1H), 7.91 (s, 1H), 7.75 (s, 1H), 4.06 (d, *J* = 6.4 Hz, 2H), 3.13 (t, *J* = 2.5 Hz, 1H); $^{13}$C NMR (100 MHz, DMSO-*d*$_6$): 168.7, 160.4, 159.1, 158.8, 157.8, 157.4, 144.2, 142.1, 141.6, 121.5, 118.4, 115.6, 81.0, 72.9, 28.2; HRMS (ESI) calcd for $C_{15}H_{12}N_7O_3S_3$ $[M+H]^+$: 434.0158; Found: 434.0157.

**Synthesis of azide fragments (2a-k)**

**Synthesis of azide 2a:** Sodium azide (2.47 g, 38 mmol) was added to the solution of *N*, *N´*-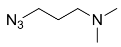 dimethyl (3-chloropropyl) amine hydrochloride (3 g, 18.9 mmol) in 40 mL water. Then the reaction mixture was heated at 70 °C for 5 h. After cooling to ambient temperature of KOH was added to the reaction mixture and extracted 3 times with diethyl ether. Removal of the diethyl ether at 0 °C under reduced pressure gave crude **2a** as a colourless liquid (1.14 g, 47%), which was directly used for copper (I) catalysed cycloaddition without any purification.

**Synthesis of the azides 2b-k:** Synthesis of azides **2b-k** has been reported previously.[5]

[5] P. Saha, D. Panda, D. Müller, A. Maity, H. Schwalbe, J. Dash *Chem. Sci.* **2020**, 11, 2058–2067.

# 4.0 NMR spectra of alkyne fragments

# $^{1}$H and $^{13}$C NMR of compound 18:

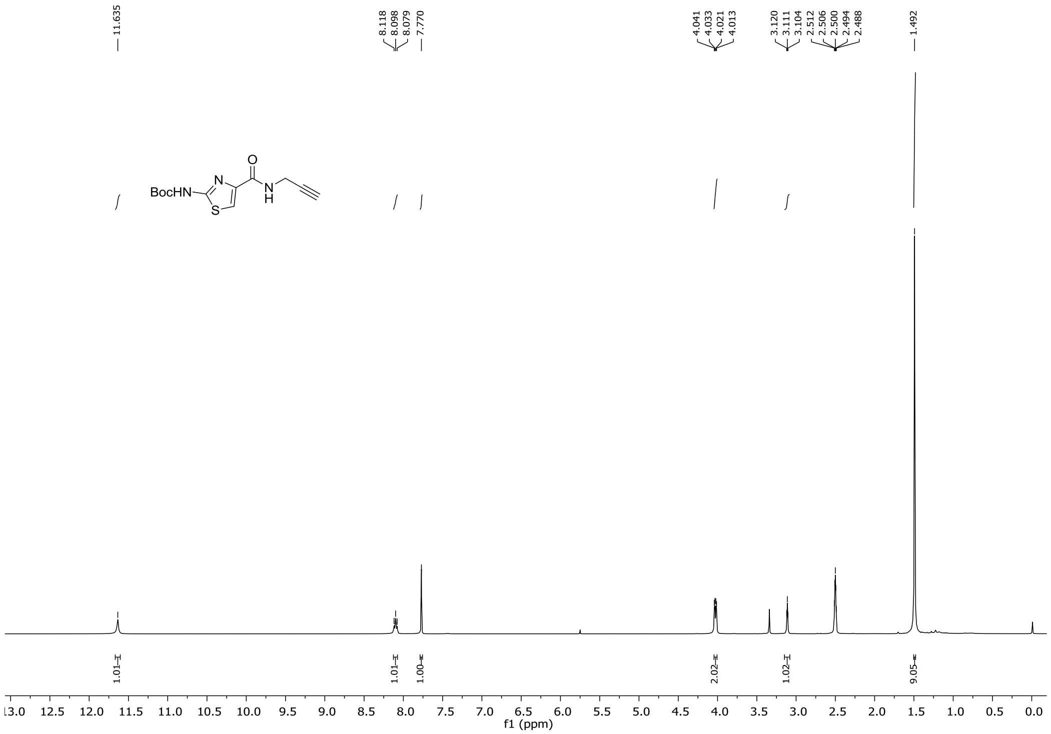


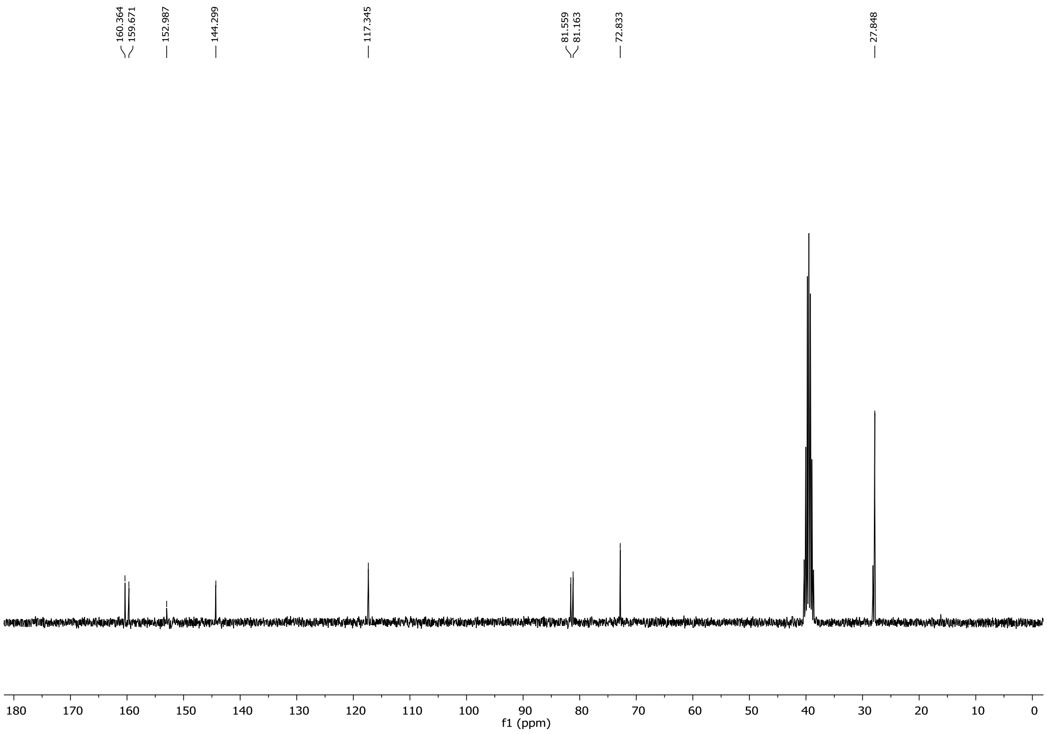

## $^{1}$H and $^{13}$C NMR of compound 19:

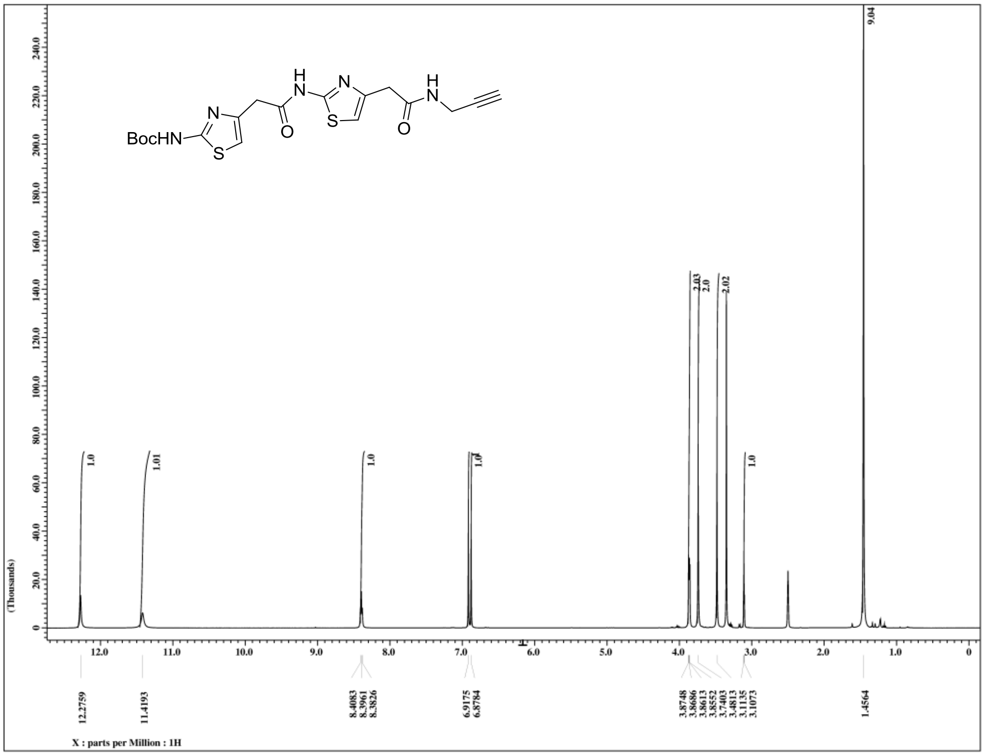


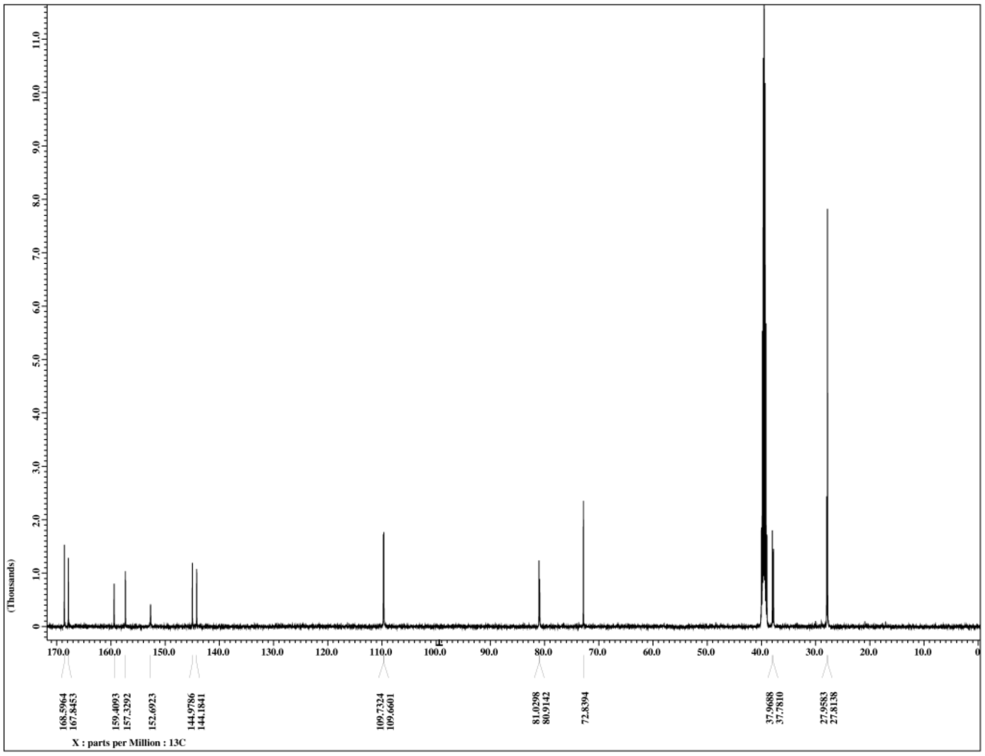

# $^{1}$H and $^{13}$C NMR of compound 20:

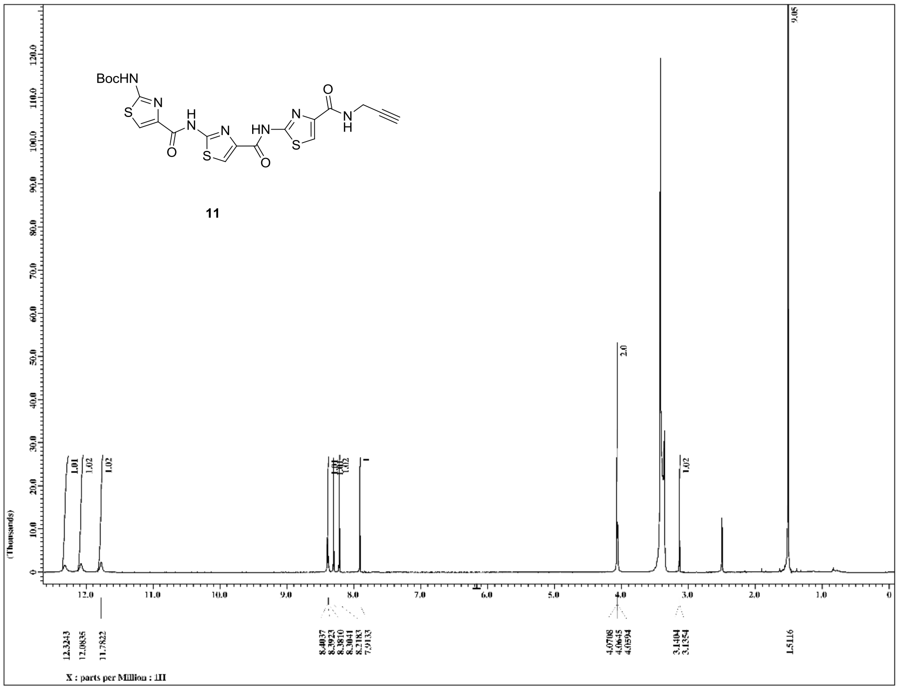


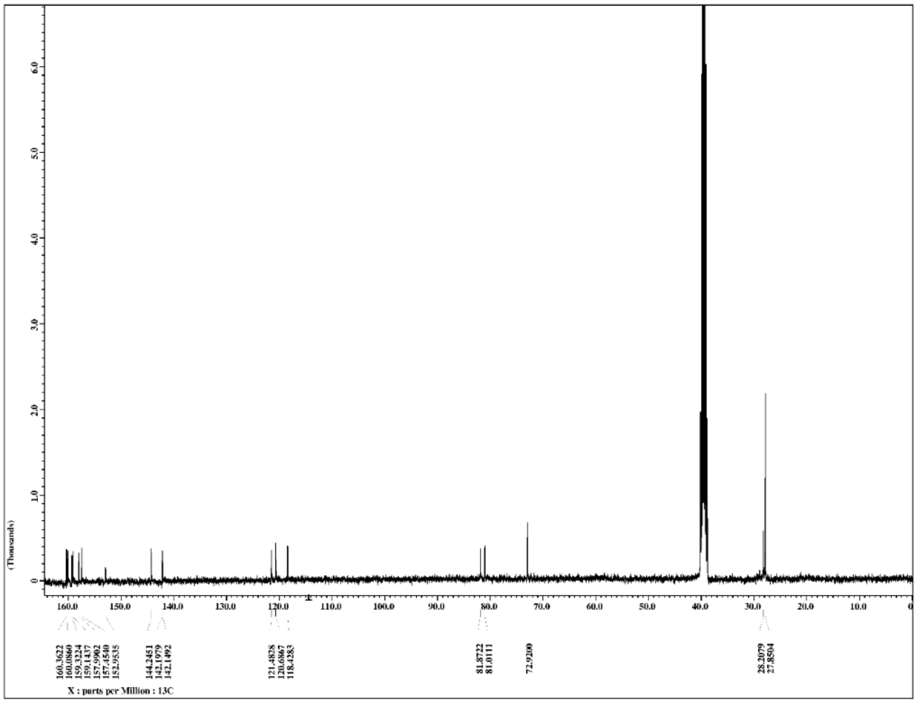

## $^{1}$H and $^{13}$C NMR of compound 1a:

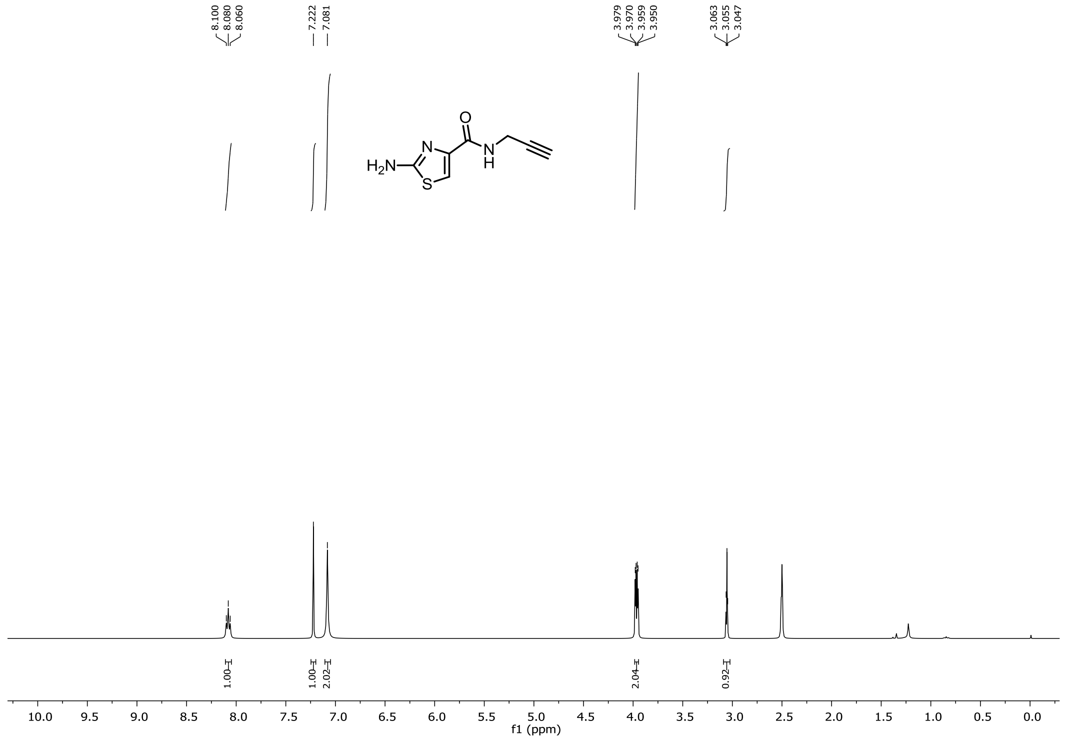


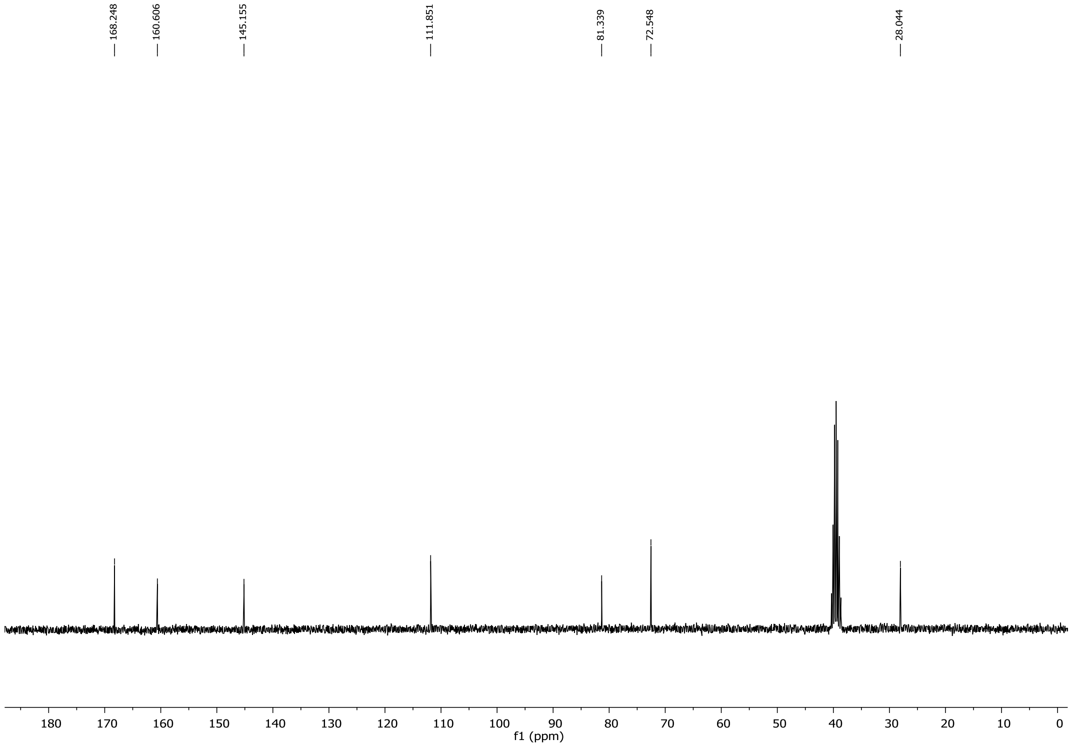

## $^{1}H$ and $^{13}C$ NMR of compound 1b:

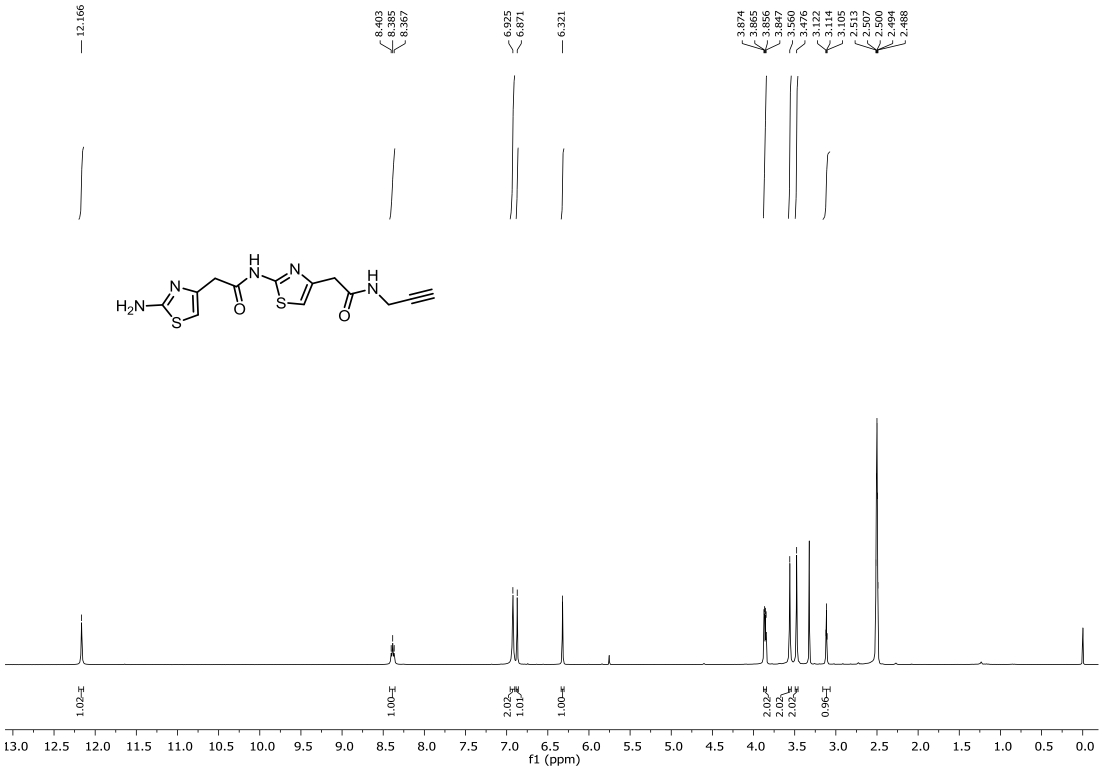


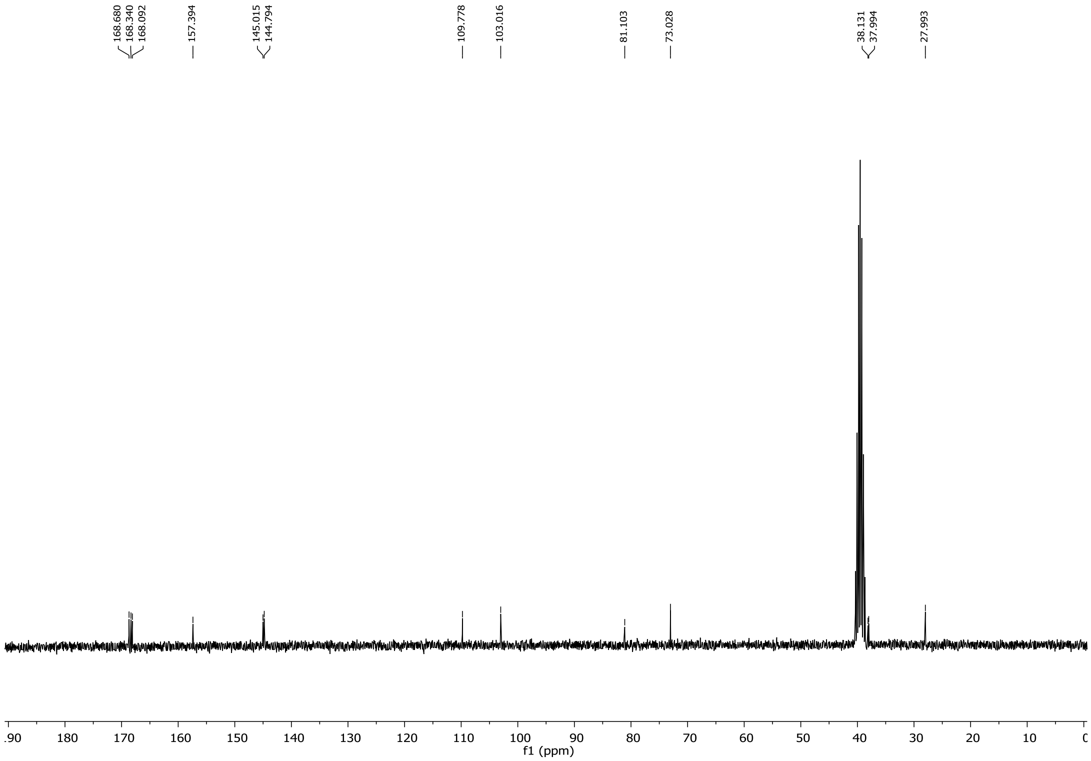

## $^{1}$H and $^{13}$C NMR of compound 1c:

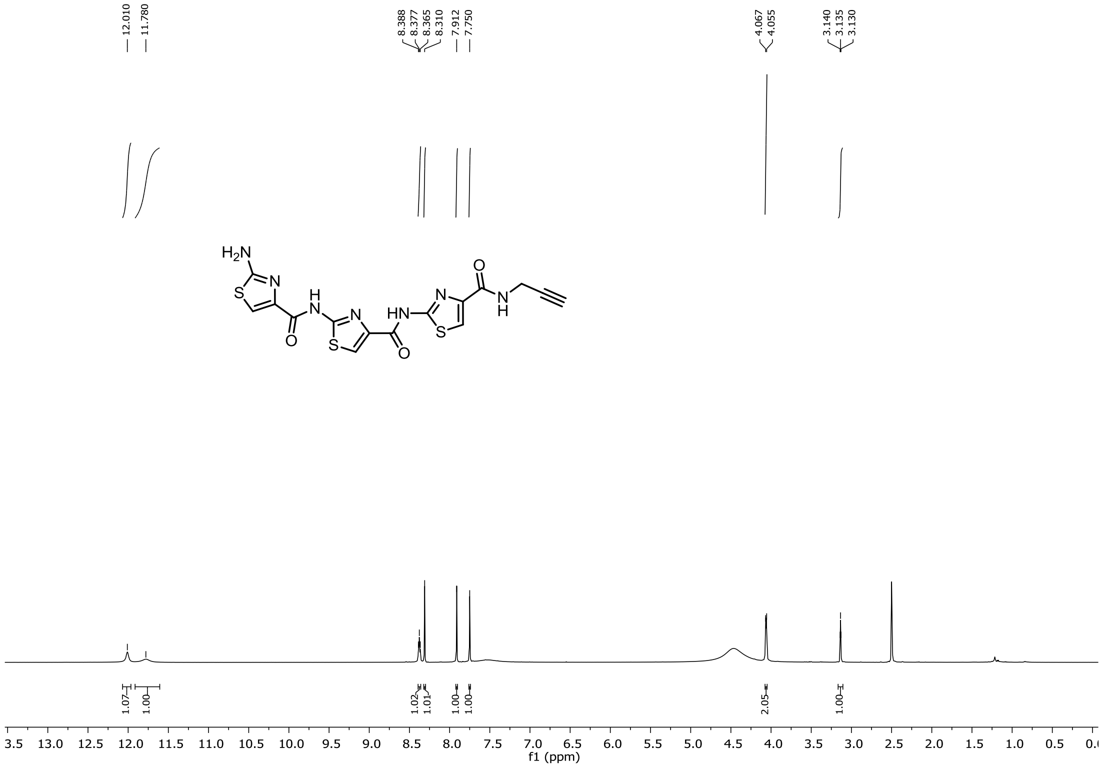


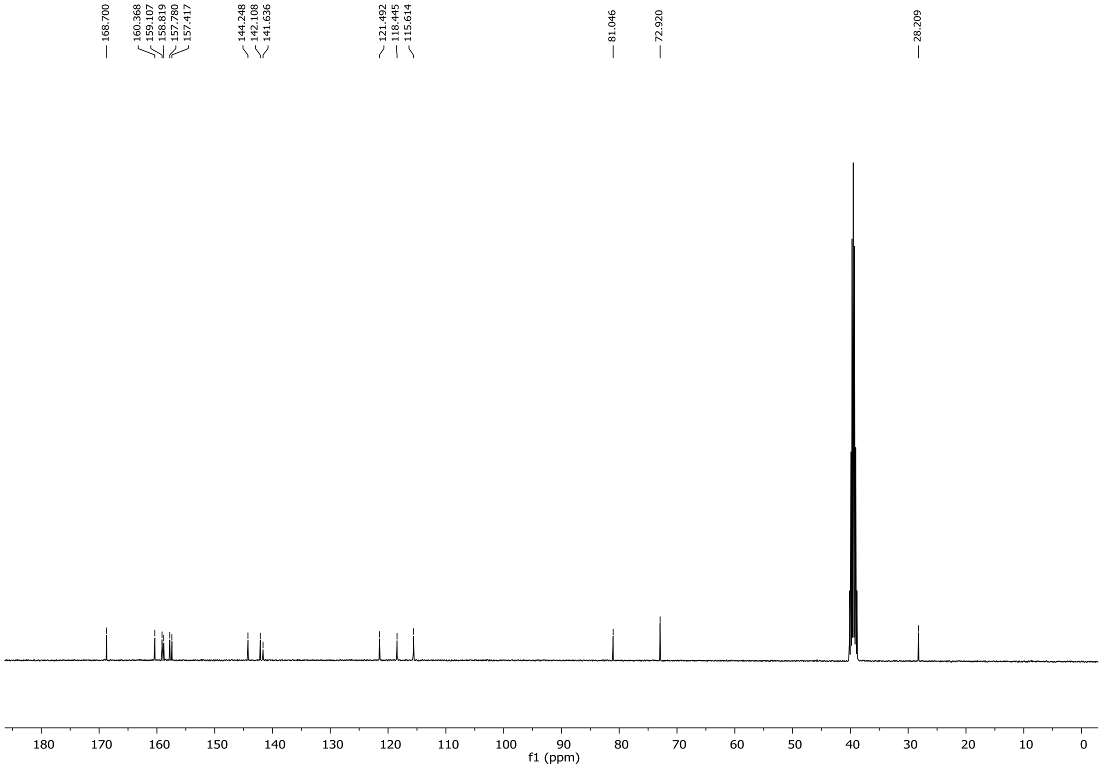

## 5.0 Oligonucleotide sequences used in *in situ* reaction

All biotin tagged/unlabeled oligomers of highest purity were purchased from Sigma-Aldrich and Eurofins. The biotin tagged oligos were pre-annealed in 20 mM sodium cacodylate, 180 mM NaCl, 10 mM $MgCl_2$ buffer, pH 7.4. Annealing was performed by heating at 85 °C for 5 min followed by rapid cooling at 4 °C to get the kinetically favoured folded structure.

**TAR RNA:** 5′ Biotin TEG -GGCAGAUCUGAGCCUGGGAGCUCUCUGCC- 3′

**TAR DNA:** 5′ Biotin TEG -GGCAGATCTGAGCCTGGGAGCTCTCTGCC- 3′

**TAR RNA w/b:** 5′ Biotin TEG -GGCAGAGAGCCUGGGAGCUCUCUGCC- 3′

## 6.0 General procedure for *in situ* cycloaddition using biotin tagged TAR RNA and control TAR RNA w/b & TAR DNA

Biotin tagged RNA and DNA were pre-annealed in 20 mM sodium cacodylate, 180 mM NaCl, 10 mM $MgCl_2$ buffer, pH 7.4. Annealing was performed by heating at 85 °C for 5 min followed by rapid cooling at 4 °C to get the kinetically favored folded structure. To a suspension of biotin tagged RNA or DNA (5 $\mu$M), azides **2a-k** (4 $\mu$M) and alkynes **1a-d** (1 $\mu$M) were added and the reaction mixture was made up to the final volume of 50 $\mu$L with buffer (20 mM sodium cacodylate, 180 mM NaCl, 10 mM $MgCl_2$ buffer, pH 7.4). The reaction mixture was incubated and continuously shaked at room temperature for 72 h. The resulting mixtures were then added with prewashed streptavidin magnetic beads and incubated for 1-2 h for non-covalent interactions of biotin with streptavidin. After incubation, the RNA or DNA along with the newly formed triazole products were separated from the reaction mixture using a magnet and washed thrice with buffer to remove the unreacted starting materials. Subsequently, the ligand bound RNA or DNA template was dispersed in 50 $\mu$L of buffer and the solution was then heated for 5 min at 85 °C. The RNA or DNA template was instantly separated by magnetic decantation and the supernatant was analyzed by HPLC. The HPLC fractions corresponding to different peaks were identified by ESI-MS spectroscopy. The HPLC analysis was performed using 5.0 $\mu$m ODS2 reverse phase column (4.6×250 mm) using 280 nm detection wavelength. Flow rate was 0.25 ml min$^{-1}$ $CH_3CN/H_2O$ (90:10) in 0.1% TFA.

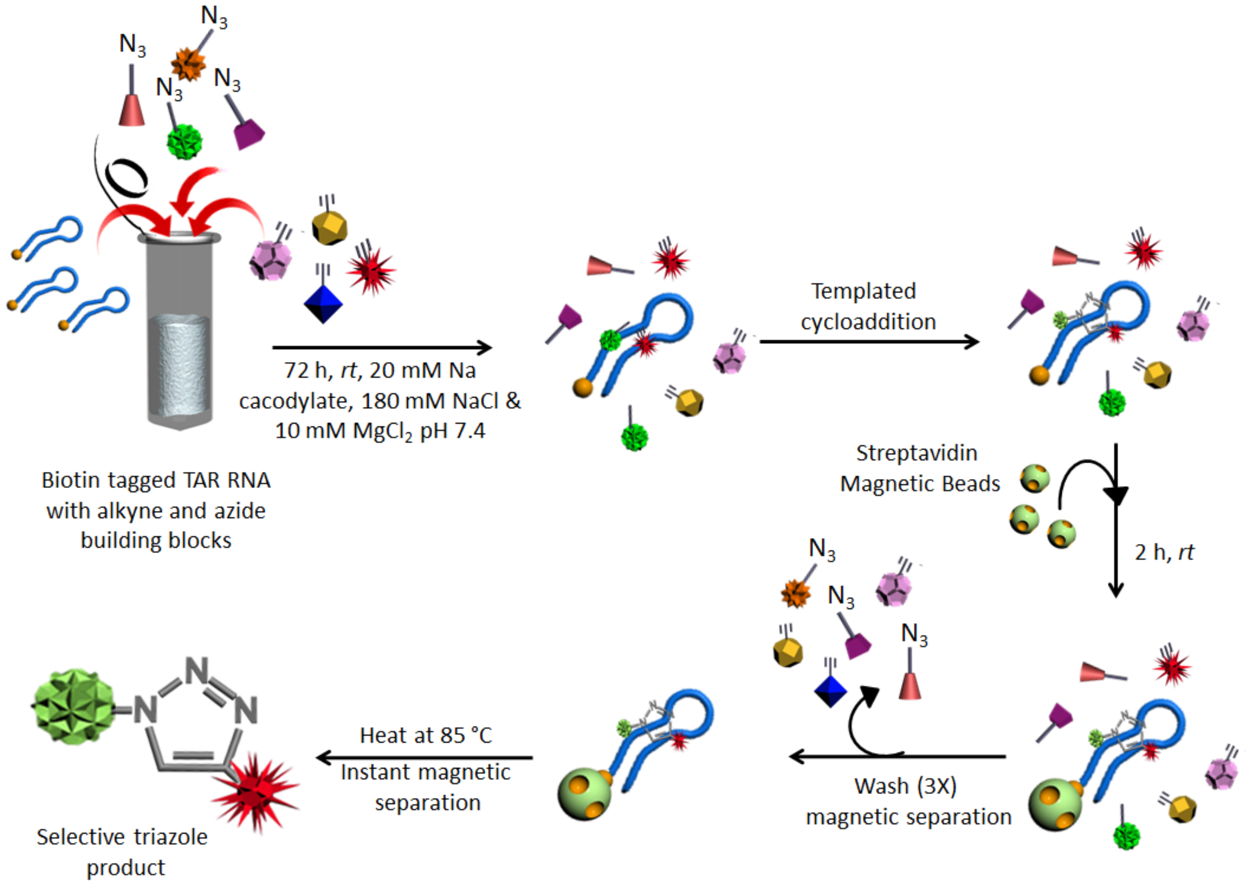


**Figure S1.** Schematic representation of the TAR RNA w/b templated cycloaddition of azide and alkyne fragments.

## 7.0 Monitoring the *in situ* cycloaddition by HPLC and ESI-MS

The isolated samples from the *in situ* cycloaddition were injected in HPLC for detection of peaks formed due to triazole products. HPLC fractions corresponding to different peaks were further collected and identified by ESI-MS spectroscopy.

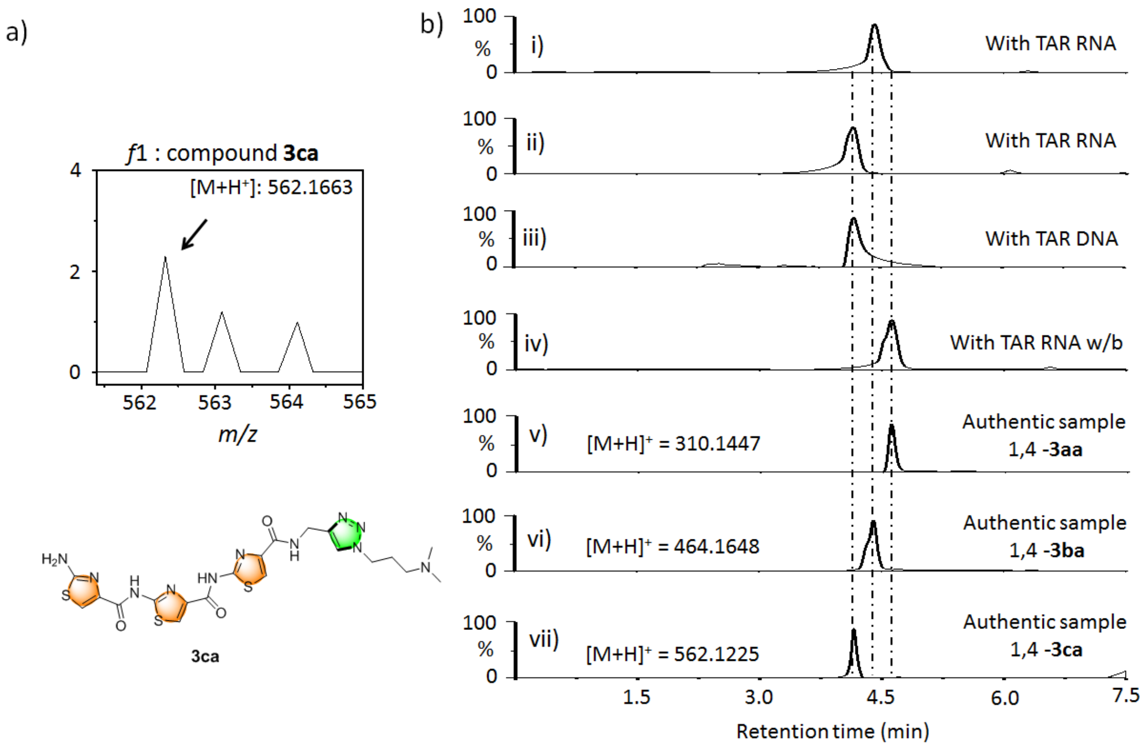


**Figure S2. (a)** Observed ESI-MS analysis of fraction *f*1 (hit triazole **3ca**) obtained with control TAR DNA. **(b)** HPLC chromatograms of (i) alkyne **1b** (1 $\mu$M) and azides **2a-k** (4 $\mu$M) with TAR RNA (5 $\mu$M), (ii) alkyne **1c** (1 $\mu$M) and azides **2a-k** (4 $\mu$M) with TAR RNA (5 $\mu$M), (iii) alkyne **1c** (1 $\mu$M) and azides **2a-k** (4 $\mu$M) with TAR DNA (5 $\mu$M), (iv) alkyne **1a** (1 $\mu$M) and azides **2a-k** (4 $\mu$M) with TAR RNA w/b (5 $\mu$M) and Authentic samples of (v) anti-**3aa**, (vi) anti-**3ba** and (vii) anti-**3ca**.

## 8.0 Relative yield of triazole leads

The yields of lead *anti*-triazoles were determined by performing time dependent cycloaddition of corresponding alkyne and azide fragments in the presence of RNA or DNA templates. Alkyne **1b** reacted with azide **2a** in the presence of TAR RNA. Similarly, alkyne **1c** reacted with azide **2a** in the presence of TAR RNA as well as TAR DNA and alkyne **1a** reacted with azide **2a** in the presence of TAR RNA w/b.

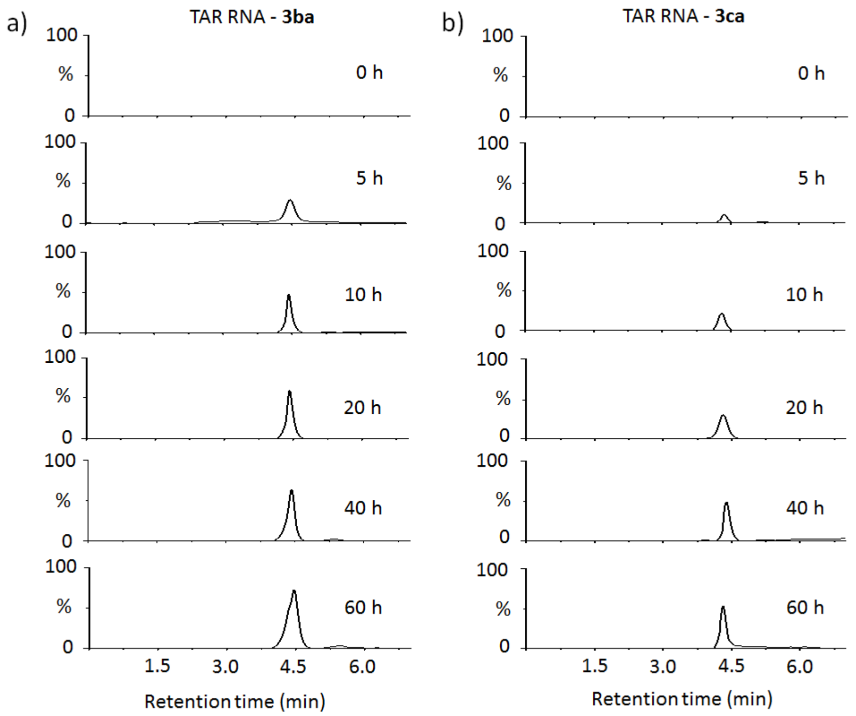


**Figure S3.** HPLC chromatogram of time dependent *in situ* cycloaddition of compounds **(a) 3ba** and **(b) 3ca** in the presence of the TAR RNA.

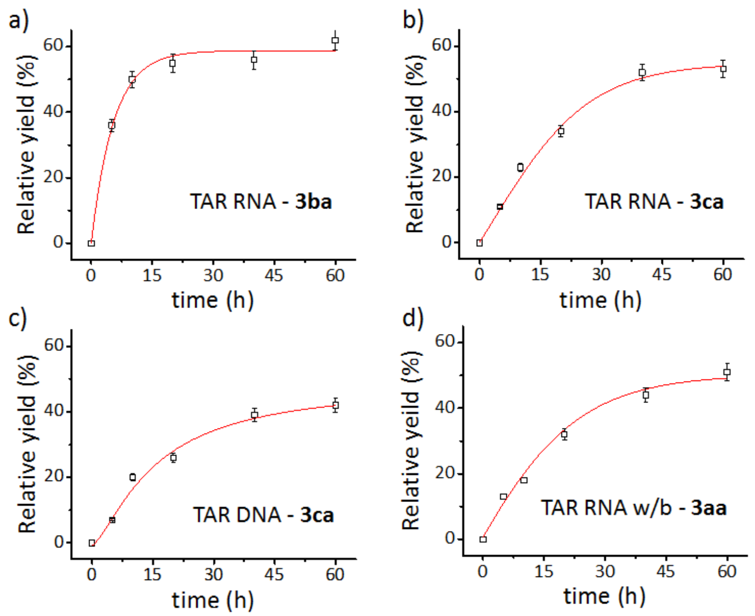


**Figure S4.** Relative yield of **(a) 3ba** with TAR RNA, **(b) 3ca** with TAR RNA, **(c) 3ca** with TAR DNA and **(d) 3aa** with TAR RNA w/b with individual respective alkynes and azides.

## 9.0 Synthesis of thiazole peptidomimetics

**Synthesis of thiazole peptide 3aa:** The Cu(I) catalyzed cycloaddition of alkyne **18** with azide **2a** provided the click product **21** in 91% yield. Subsequent Boc-deprotection using trifluoroacetic acid provided the triazole linked thiazole peptide **3aa** in high yield.

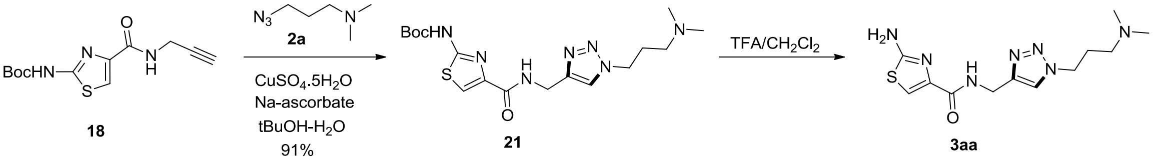


**Scheme S4.** Synthesis of thiazole peptide **3aa**.

**Synthesis of triazole containing thiazole peptide 21:** Using general procedure **GP-5**, alkyne **18**

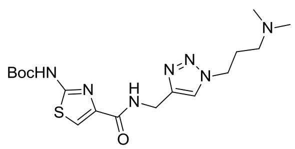

(100 mg, 0.355 mmol), copper(II) sulphate pentahydrate (9 mg, 0.0355 mmol), sodium ascorbate (14 mg, 0.071 mmol) and azide **2a** (454 mg, 3.55 mmol) in *t*BuOH/$H_2O$ (2 mL) provided the desired compound **21** (132 mg, 91%) as a colorless liquid; $^1$H NMR (300 MHz, DMSO-$d_6$): 11.62 (s, 1H), 8.13 (s, 1H), 7.96 (s, 1H), 7.76 (s, 1H), 4.50 (d, $J$ = 5.8 Hz, 2H), 4.33 (t, $J$ = 7.2 Hz, 2H), 2.16 (t, $J$ = 6.9 Hz, 2H), 2.10 (s, 6H), 1.96–1.86 (m, 2H), 1.49 (s, 9H); $^{13}$C NMR (75 MHz, DMSO-$d_6$): 160.5, 159.7, 144.5, 144.3, 122.9, 117.1, 81.5, 55.6, 47.4, 45.0, 34.4, 27.8, 27.7; HRMS (ESI) calcd for $C_{17}H_{28}N_7O_3S$ $[M+H]^+$: 410.1969; Found: 410.1968.

**Synthesis of thiazole peptide 3aa:** Using general procedure **GP-4**, Boc protected thiazole

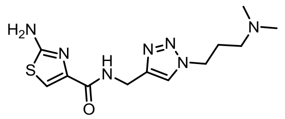

peptide **21** (100 mg, 0.15 mmol) was treated with trifluoroacetic acid (1 mL) in $CH_2Cl_2$ (1 mL) to provide the corresponding thiazole peptide **3aa** (89 mg, 89%) as a white solid; $^1$H NMR (300 MHz, DMSO-$d_6$): 8.13 (t, $J$ = 6.0 Hz, 1H), 7.91 (s, 1H), 7.21 (s, 1H), 7.10 (s, 2H), 4.45 (d, $J$ = 5.9 Hz, 2H), 4.32 (t, $J$ = 7.1 Hz, 2H), 2.16 (t, $J$ = 6.9 Hz, 2H), 2.10 (s, 6H), 1.95-1.85 (m, 2H); $^{13}$C NMR (75 MHz, DMSO-$d_6$): 168.2, 160.8, 145.4, 144.7, 122.8, 111.5, 55.6, 47.5, 45.0, 34.3, 27.8; HRMS (ESI) calcd for $C_{12}H_{20}N_7OS$ $[M+H]^+$: 310.1969; Found: 310.1447. HPLC purity: 98%. The HPLC chromatogram of thiazole peptide **3aa** is given below.

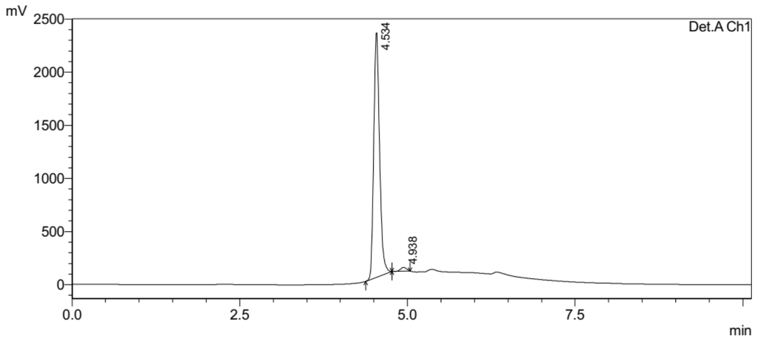


PeakTable

Detector A Ch1 280nm

| Peak# | Ret. Time | Area | Height | Area % | Height % |
|---|---|---|---|---|---|
| 1 | 4.534 | 13885253 | 2296891 | 98.414 | 98.453 |
| 2 | 4.938 | 223744 | 36088 | 1.586 | 1.547 |
| Total | | 14108997 | 2332979 | 100.000 | 100.000 |

**Synthesis of thiazole peptide 3ba:** The Cu(I) catalyzed cycloaddition of alkyne **19** with azide **2a** provided the click product **22** in 83% yield. Subsequent Boc-deprotection of **22** using trifluoroacetic acid provided the triazole linked thiazole peptide **3ba** in high yield.

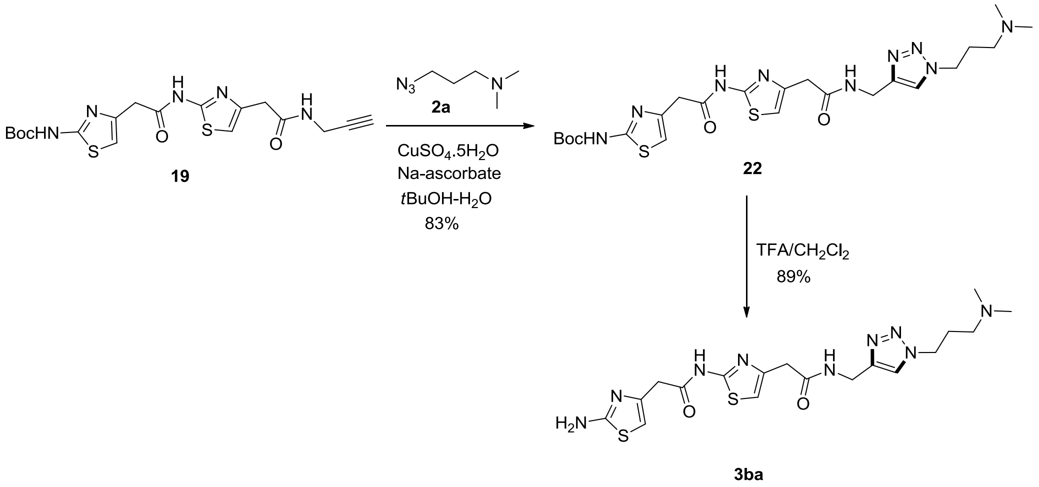


**Scheme S5.** Synthesis of thiazole peptide **3ba**.

**Synthesis of thiazole peptide 22:** Using general procedure **GP-5**, alkyne **19** (100 mg, 0.23 mmol), copper(II) sulphate pentahydrate (5.7 mg, 0.023 mmol), sodium ascorbate (8.7 mg, 0.046 mmol) and azide **2a** (295 mg, 2.3 mmol) in *t*BuOH/$H_2O$ (2 mL) provided the

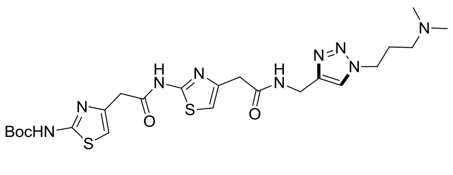

desired compound **22** (107 mg, 83%) as an off-white solid; $^{1}$H NMR (400 MHz, $CDCl_3$): 7.93 (1H, s), 7.51 (1H, s), 6.71 (1H, s), 6.65 (1H, s), 4.41 (2H, d, *J* = 5.3 Hz), 4.35 (2H, t, *J* = 6.8 Hz), 3.82 (2H, s), 3.56 (2H, s), 2.25 (2H, t, *J* = 6.8 Hz), 2.19 (6H, s), 2.05-1.98 (2H, m), 1.51 (9H, s); $^{13}$C NMR (100 MHz, $CDCl_3$): 169.8, 167.5, 161.7, 158.6, 152.7, 144.6, 144.1, 142.7, 122.7, 110.8, 110.3, 82.5, 55.9, 48.3, 45.3, 38.9, 38.5, 34.9, 28.3, 28.0; HRMS (ESI) calcd for $C_{23}H_{34}N_9O_4S_2$ $[M+H]^+$: 564.2170; Found: 564.2172.

**Synthesis of thiazole peptide 3ba:** Using general procedure **GP-4**, Boc protected thiazole peptide **22** (80 mg, 0.142 mmol) treated with trifluoroacetic acid (1 mL) in $CH_2Cl_2$ (1 mL) to provide the corresponding thiazole peptide **3ba** (58 mg, 89%) as a white solid; $^{1}$H NMR (400 MHz, DMSO-$d_6$): 12.19 (1H, $s_{br}$), 8.45 (1H, t, *J* = 5.4 Hz), 7.89 (1H, s), 6.93 (2H, s), 6.87 (1H, s), 6.32 (1H, s), 4.33 (2H, t, *J* = 6.8 Hz), 4.30 (2H, d, *J* = 5.8 Hz), 3.56 (2H, s), 3.49 (2H, s), 2.16 (2H, t, *J* = 6.8 Hz), 2.10 (6H, s), 1.94-1.87 (2H, m); $^{13}$C NMR (100 MHz, DMSO-$d_6$): 168.9, 168.4, 168.1, 157.4, 145.3, 144.8, 144.6, 122.9, 109.6, 103.1, 55.6, 47.4, 45.0, 38.2, 38.1, 34.4, 27.8; HRMS (ESI) calcd for $C_{18}H_{26}N_9O_2S_2$ $[M+H]^+$: 464.1645; Found: 464.1648. HPLC purity: 99%. The HPLC chromatogram of thiazole peptide **3ba** is given below.

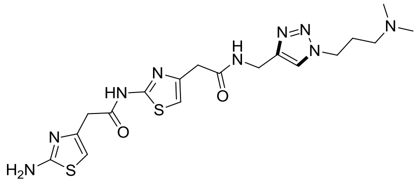

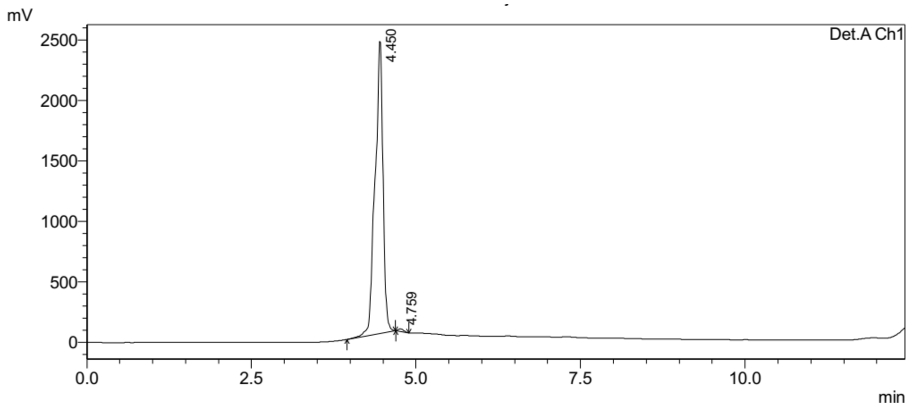

PeakTable

Detector A Ch1 280nm

| Peak# | Ret. Time | Area | Height | Area % | Height % |
|---|---|---|---|---|---|
| 1 | 4.450 | 20406211 | 2414730 | 99.406 | 99.131 |
| 2 | 4.759 | 121988 | 21174 | 0.594 | 0.869 |
| Total | | 20528199 | 2435905 | 100.000 | 100.000 |

**Synthesis of thiazole peptide 3ca:** The Cu(I) catalyzed cycloaddition of alkyne **20** with azide **2a** provided the click product **23** in 90% yield. The Boc-deprotection of **23** using trifluoroacetic acid provided the triazole linked thiazole peptide **3ca** in high yield. Peptide **3ca** was obtained as its TFA salt.

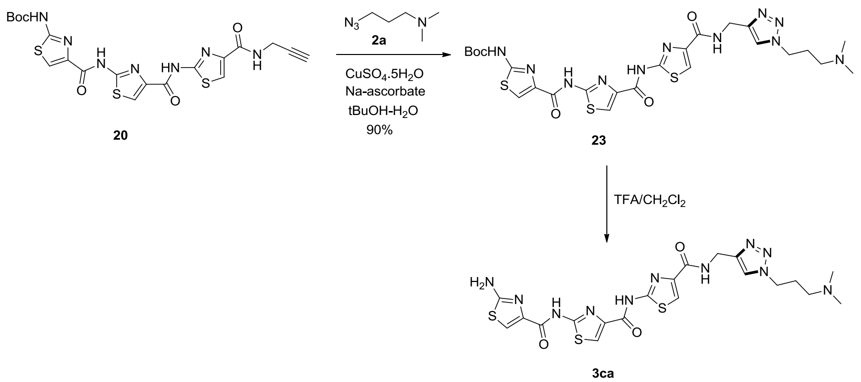


**Scheme S6.** Synthesis of thiazole peptide **3ca**.

**Synthesis of triazole containing thiazole peptide 22:** Using general procedure **GP-5**, alkyne **20** (100 mg, 0.187 mmol), copper(II) sulphate pentahydrate (7 mg, 0.0187 mmol) and sodium ascorbate (10 mg, 0.0374 mmol) and azide **2a** (240 mg, 1.87 mmol) in *t*BuOH/$H_2O$ (2 mL) provided the desired compound **23** (112 mg, 90 %) as a white solid;

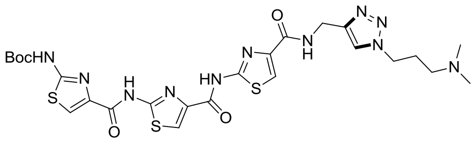


$^1$H NMR (500 MHz, DMSO-d$_6$): 8.44 (1H, t, *J* = 5.8 Hz), 8.26 (1H, s), 8.18 (1H, s), 7.98 (1H, s), 7.89 (1H, s), 4.53 (2H, d, *J* = 5.8 Hz), 4.34 (2H, t, *J* = 7.5 Hz), 2.22 (2H, t, *J* = 6.7 Hz), 2.14 (6H, s), 1.96-1.90 (2H, m), 1.51 (9H, s); $^{13}$C NMR (100 MHz, DMSO-d$_6$): 160.5, 160.0, 159.6, 159.3, 158.4, 157.6, 152.9, 144.4, 144.3, 142.6, 142.2, 122.9, 121.3, 120.5, 118.1, 81.9, 55.5, 47.4, 44.9, 34.4, 27.9, 27.6; HRMS (ESI) calcd for $C_{25}H_{32}N_{11}O_5S_3$ [M+H]$^+$: 662.1745; Found: 662.1743.

**Synthesis of triazole containing thiazole peptide 3ca:** Thiazole peptide **23** (100 mg, 0.15 mmol) was dissolved in $CH_2Cl_2$ (1 mL) and cooled to 0 °C. 1 mL trifluoroacetic acid (equal amount as the solvent) was added and the solution was allowed to warm to room temperature. The reaction mixture was stirred for about 3-4 hour at room temperature until starting material was fully consumed. Reaction was monitored by TLC. After completion of the reaction, the solvent was removed in vacuo and the residue was washed with ether. The solid residue was dried under vacuum to provide the corresponding amine free triazole containing thiazole peptide **3ca** (89 mg, 89%) as a white solid; $^1$H NMR (500 MHz, $D_2O$/DMSO-$d_6$= 4:1): 7.86 (1H, s), 7.71 (1H, s), 7.56 (1H, s), 7.45 (1H, s), 4.38-4.35 (4H, merged), 3.01 (2H, t, *J* = 6.7 Hz), 2.71 (6H, s), 2.21-2.15 (2H, m); $^{13}$C NMR (125 MHz, $D_2O$/DMSO-$d_6$= 4:1): 170.7, 163.5, 162.7 (q, *J* = 33.7 Hz), 160.7, 158.9, 158.8, 146.0, 144.6, 142.8, 136.1, 125.3, 123.9, 120.7, 117.7 (q, *J* = 290.7 Hz), 116.9, 114.2, 55.7, 48.3, 44.0, 35.4, 26.0; peaks for trifluoroacetate salt were observed at 162.7 (q, *J* = 33.7 Hz), 117.7 (q, *J* = 290.7 Hz) in $^{13}$C NMR; HRMS (ESI) calcd for $C_{20}H_{24}N_{11}O_3S_3$ $[M+H]^+$: 562.1220; Found: 562.1225. HPLC purity: 95%. The HPLC chromatogram of thiazole peptide **3ca** is given below.

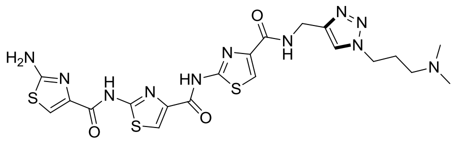

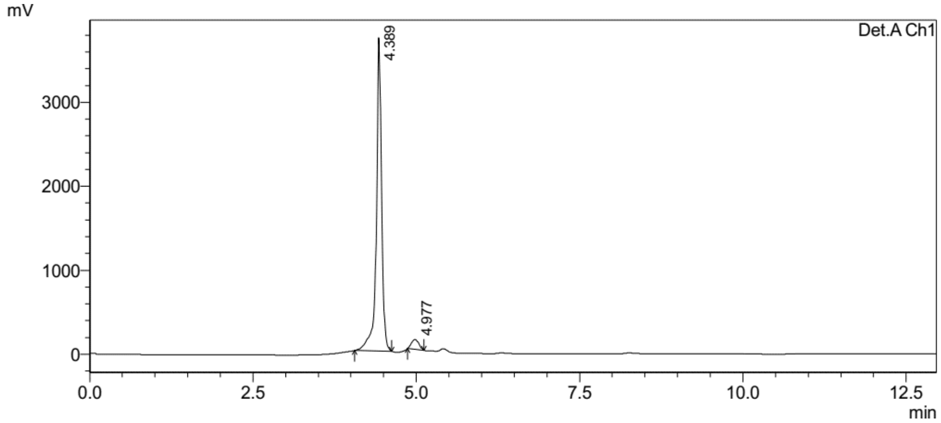


PeakTable

Detector A Ch1 280nm

| Peak# | Ret. Time | Area | Height | Area % | Height % |
|---|---|---|---|---|---|
| 1 | 4.389 | 20735110 | 3728816 | 95.767 | 96.939 |
| 2 | 4.977 | 916593 | 117755 | 4.233 | 3.061 |
| Total | | 21651704 | 3846571 | 100.000 | 100.000 |

## 10.0 NMR spectra of all compounds

## $^{1}$H and $^{13}$C NMR of compound 21:

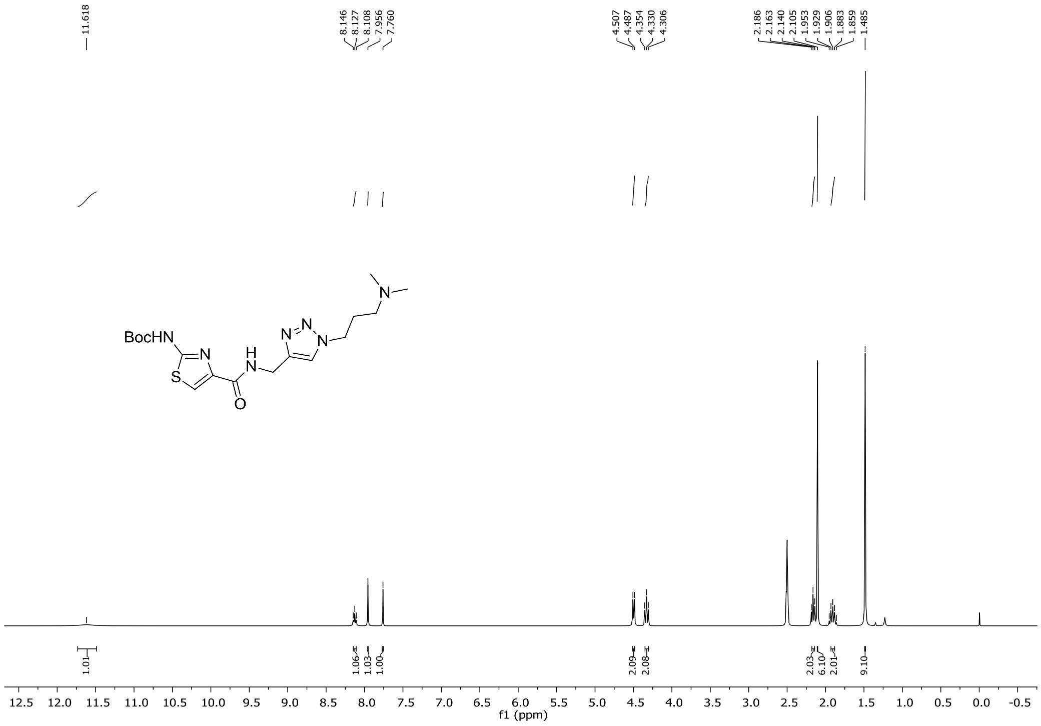


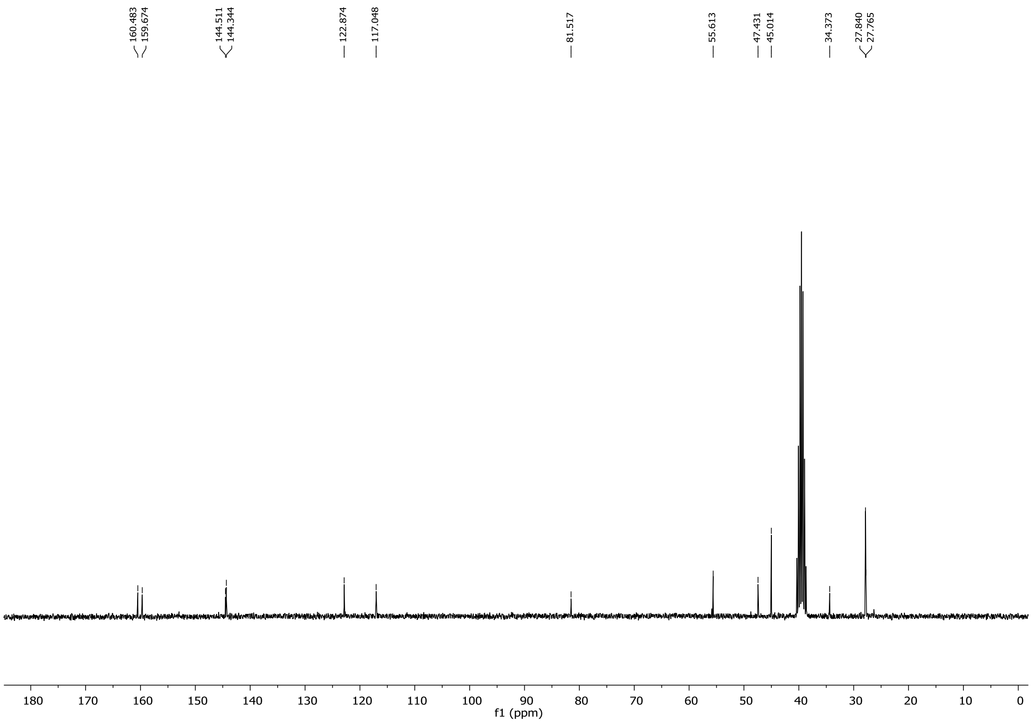

**$^{1}$H and $^{13}$C NMR of compound 1aa:**

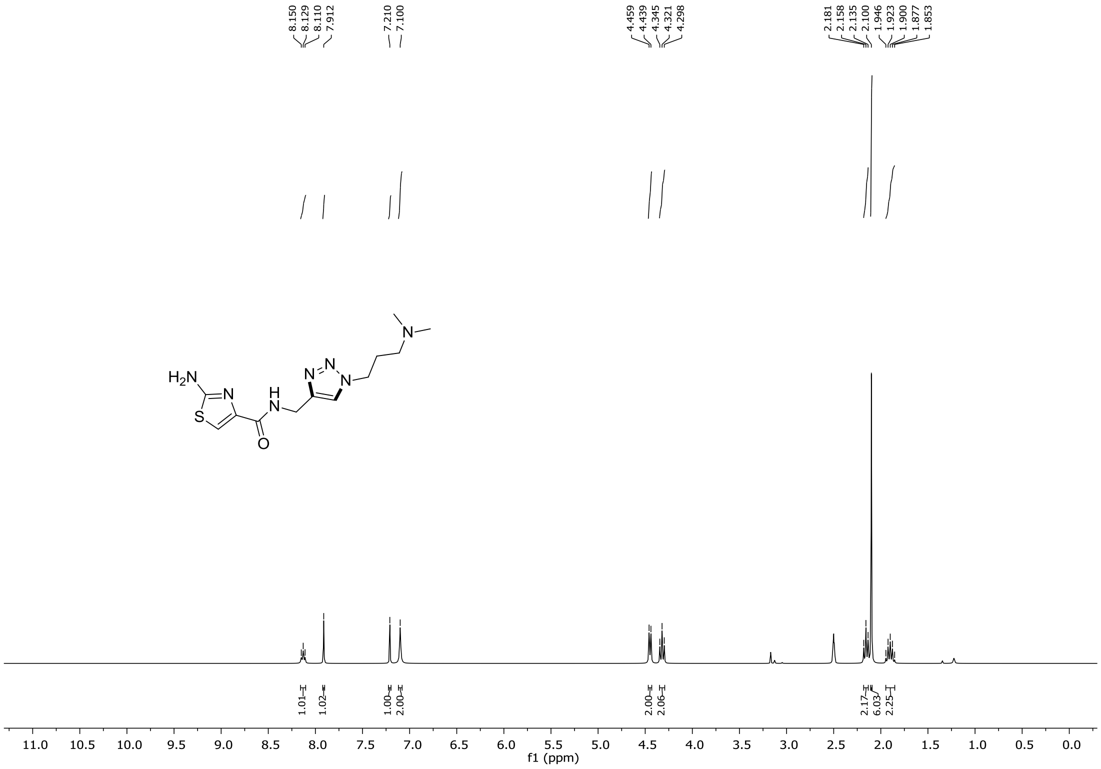

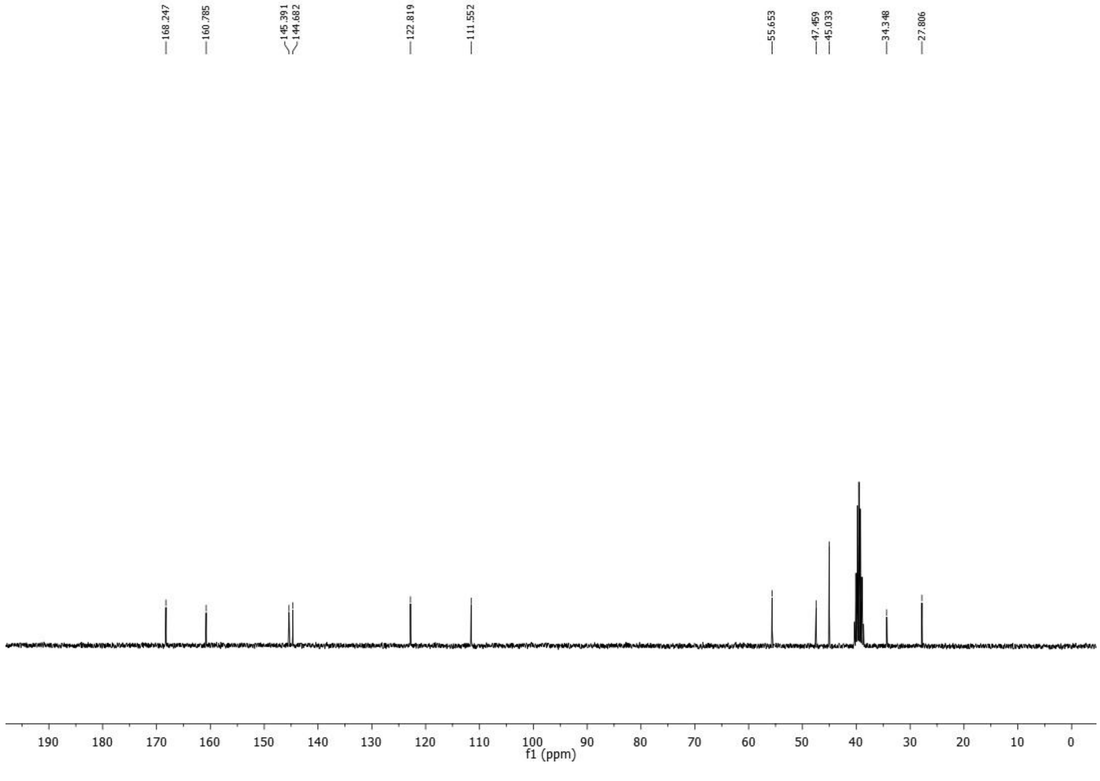

# $^{1}$H and $^{13}$C NMR of compound 22:

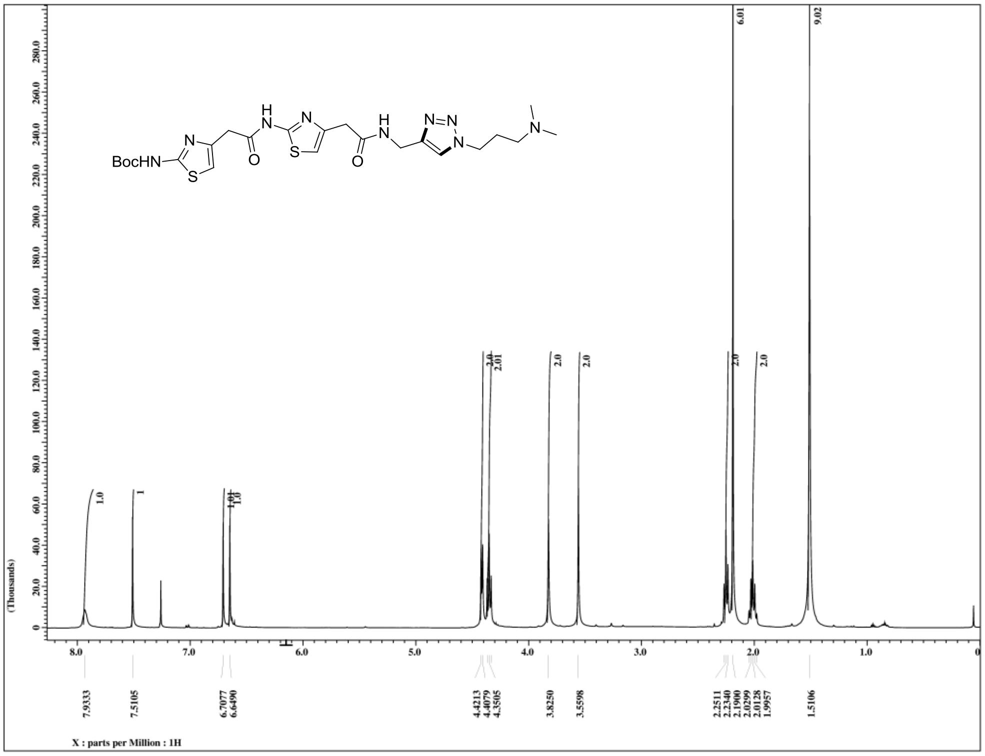

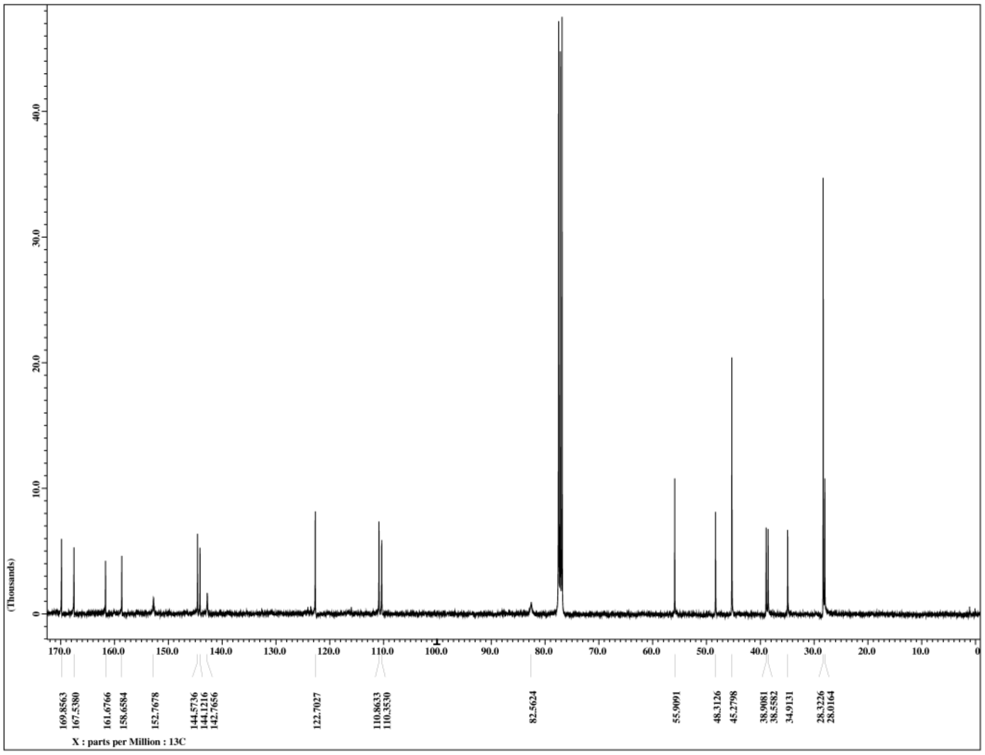

## $^{1}H$ and $^{13}C$ NMR of compound 3ba:

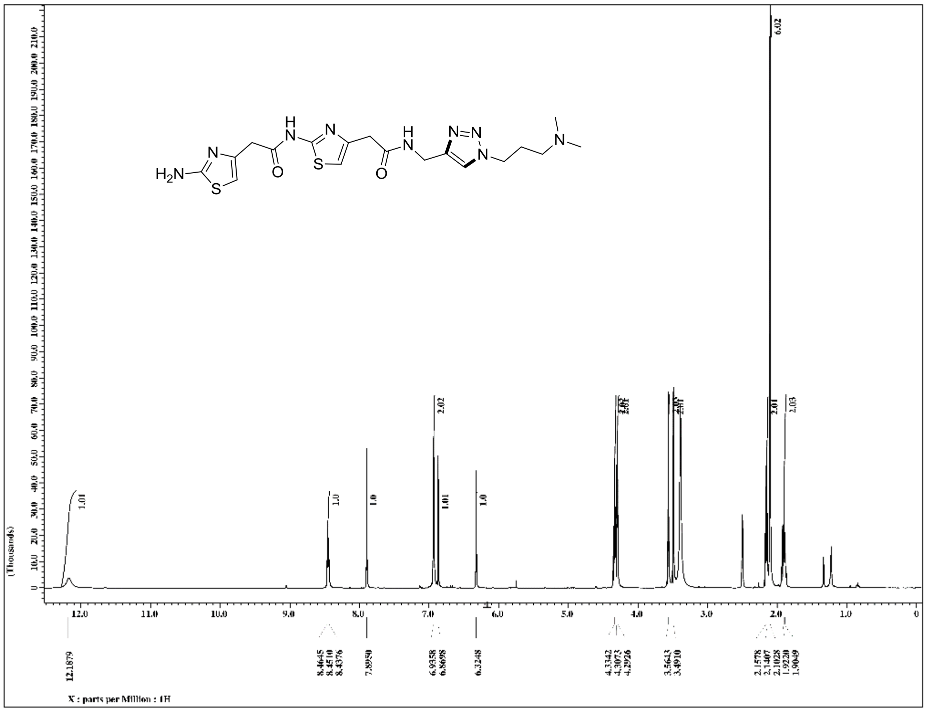


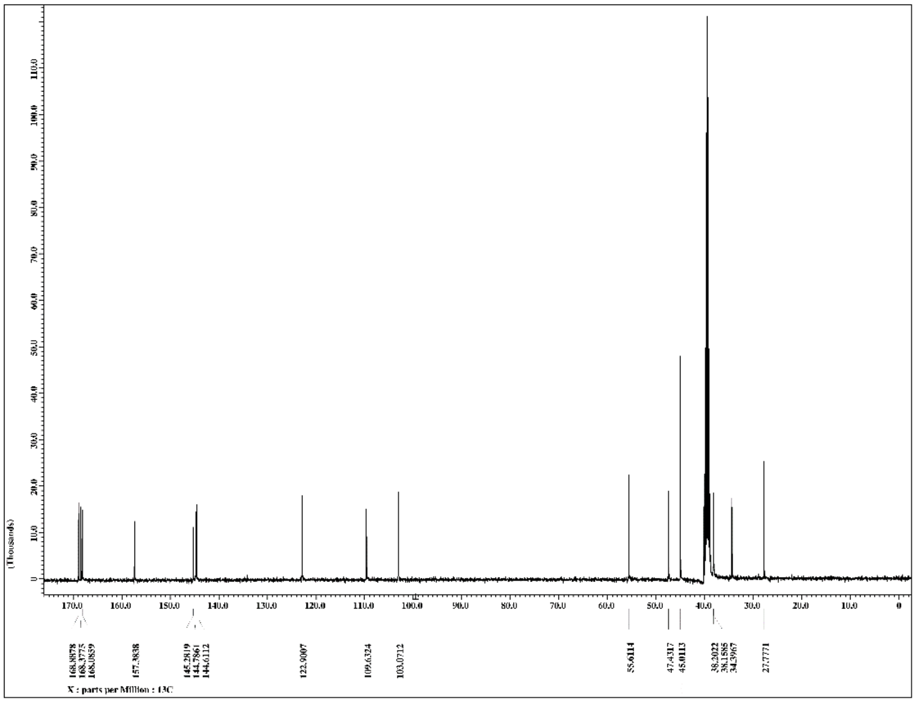

**$^{1}$H and $^{13}$C NMR of compound 23:**

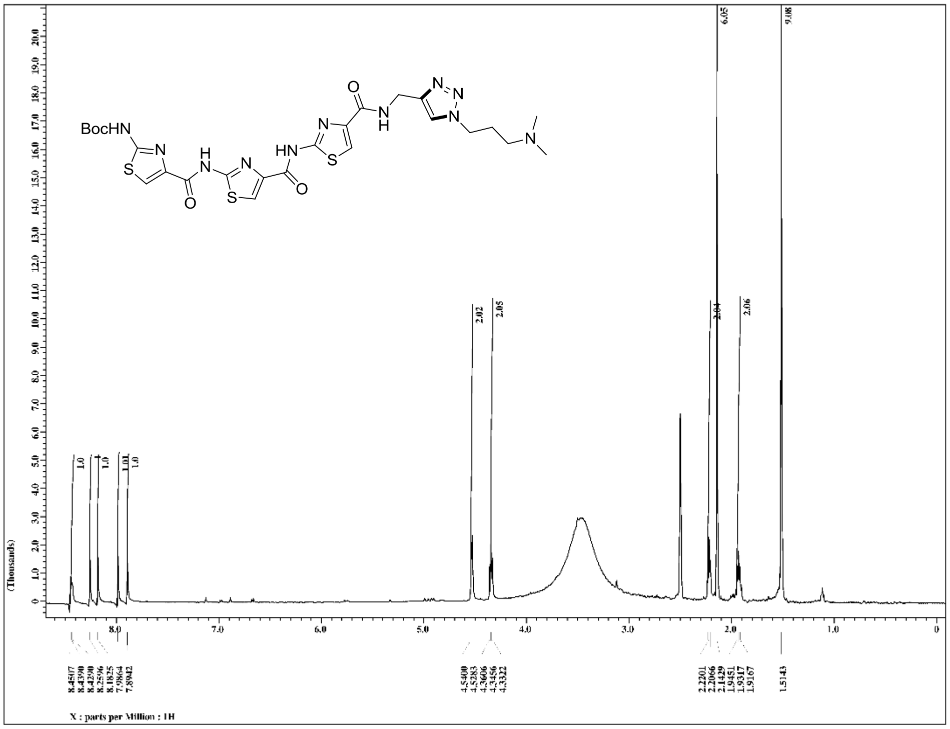


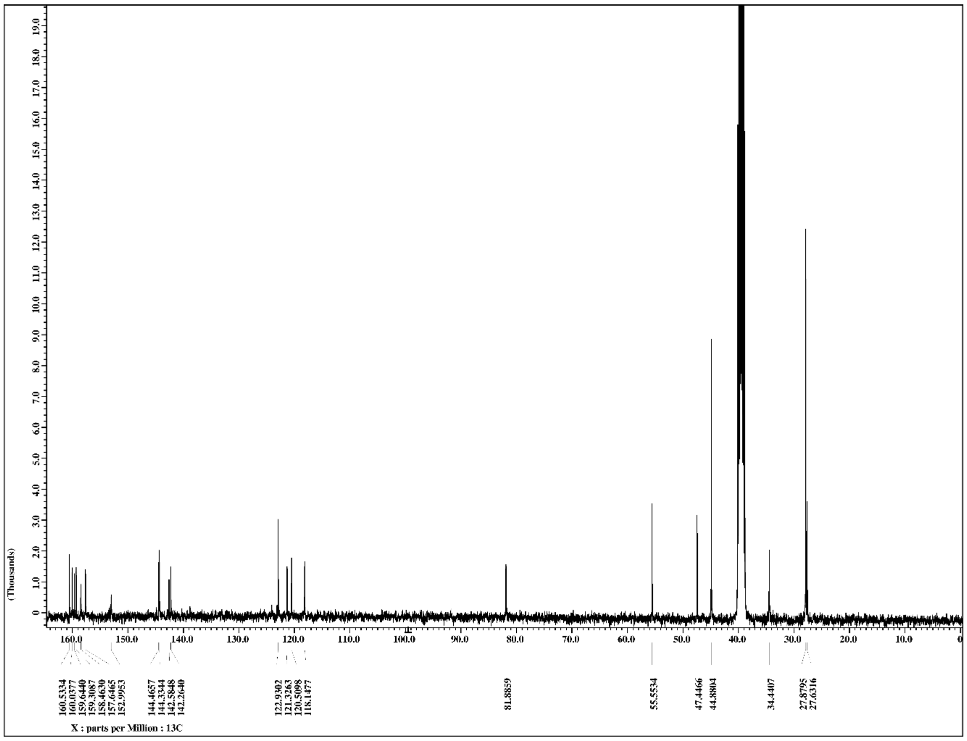

**$^{1}$H and $^{13}$C NMR of compound 3ca:**

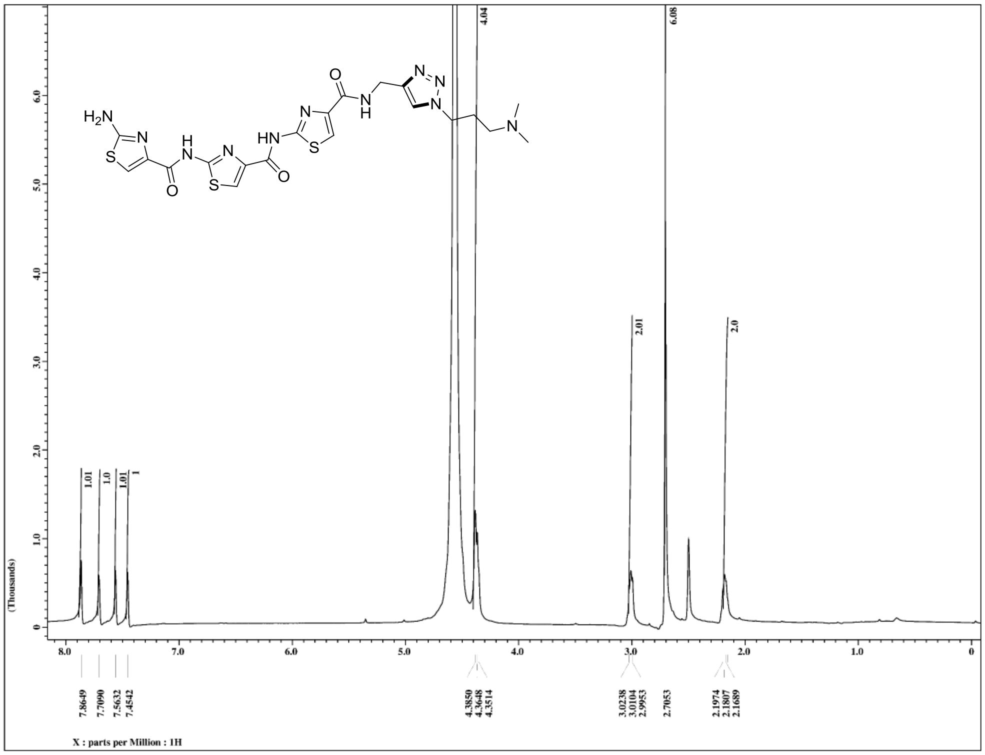

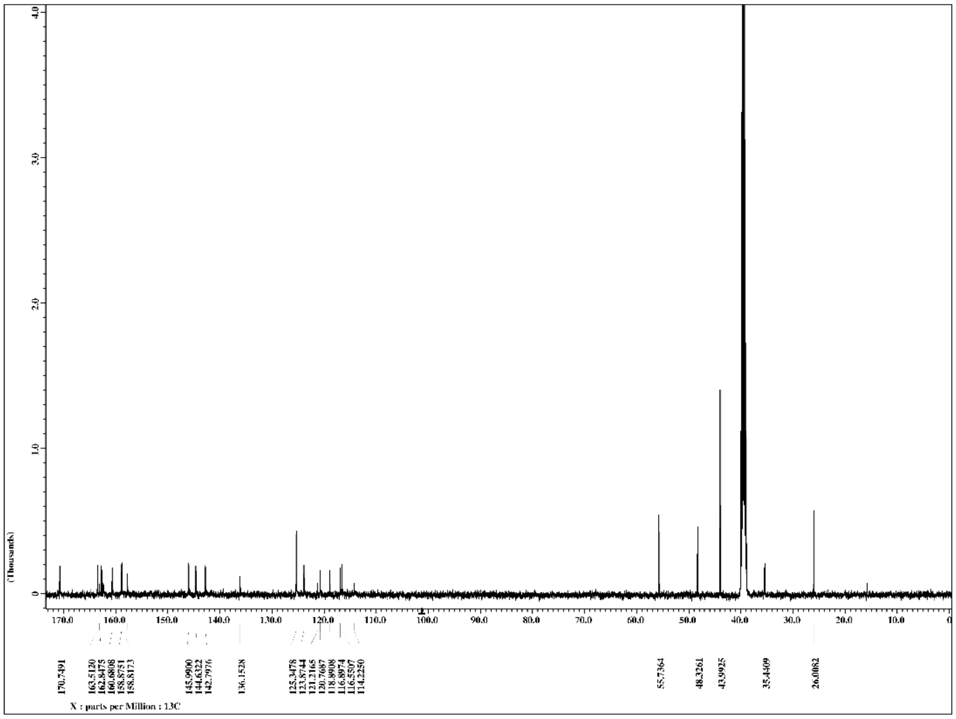

## 11.0 ITC studies

Isothermal titration calorimetry experiments were performed on a Microcal PEAQ-ITC micro-calorimeter (Malvern, USA). RNA or DNA was first annealed as mentioned previously and loaded in the experimental cell of the instrument. RNA or DNA to ligand ratio was taken as 1:10 and the titration was carried out in 20 mM sodium cacodylate, 180 mM NaCl, 10 mM $MgCl_2$ buffer, pH 7.4 at 25 ˚C. Experiments were calibrated using the same buffer loaded in the reference cell. Final analysis of the data was carried out using Originpro 8.0 (OriginLab Corp.).

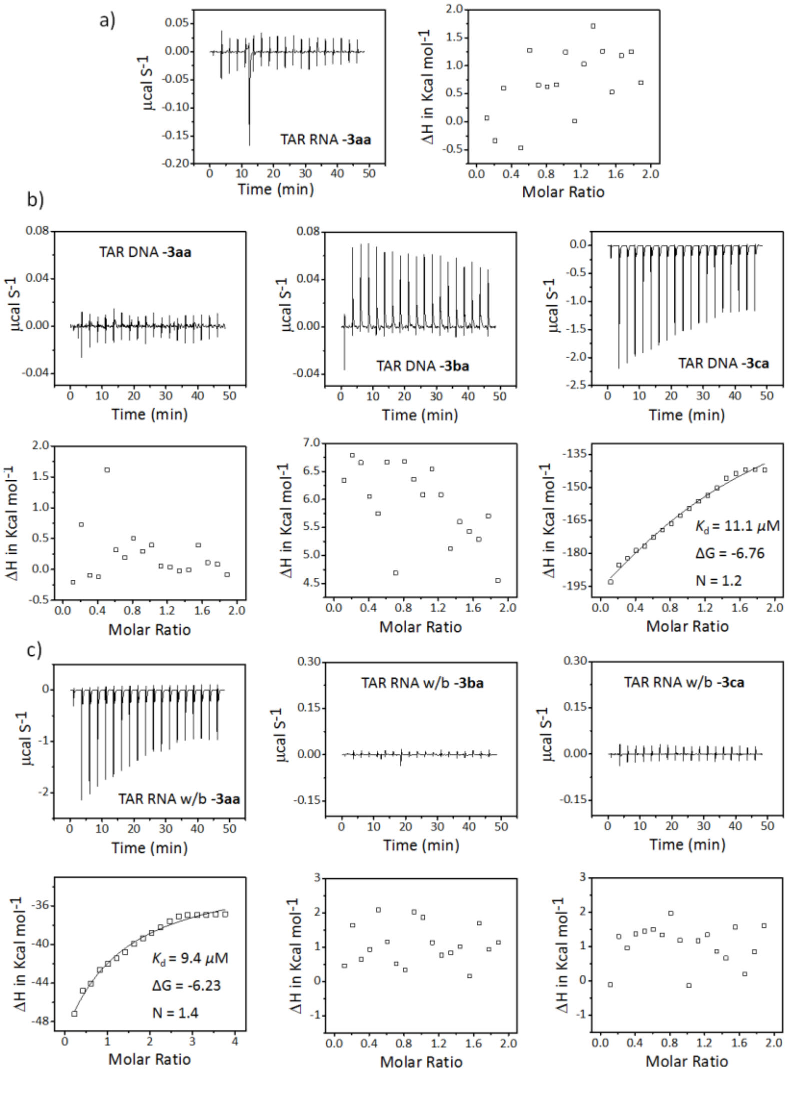


**Figure S5.** Raw heat burst curves and isotherm profiles of the integrated heat versus the ligand-RNA/DNA molar ratio obtained from ITC binding studies for the interactions of leads **(a) 3aa**

with TAR RNA, **(b) 3aa**, **3ba** and **3ca** with TAR DNA and **(c) 3aa**, **3ba** and **3ca** with TAR RNA w/b in the presence of 20mM Na cacodylate, 180 mM NaCl and 10 mM $MgCl_2$ buffer pH 7.4.

## 12.0 Fluorescence titration study

Fluorescence spectra were measured in a Horiba JobinYvon Flurolog spectrofluorimeter FL3-11 at 25 °C in thermostated cell holder using 1 mm path length micro quartz cuvette with filtered buffer (20mM Na cacodylate, 180 mM NaCl and 10 mM $MgCl_2$ buffer pH 7.4). RNA or DNA was annealed as mentioned previously and fluorescence titrations were performed with successive addition of those RNA or DNA solution into the 2 μM ligand solution. Final analysis of the data was carried out using Originpro 8.0 (OriginLab Corp.) and the dissociation constant value ($K_d$) was calculated using the Hill-1 formula:

$$F = F_0 + \frac{(F_{max} - F_0)[DNA]}{K_d + [DNA]}$$

$F$ is the fluorescence intensity, $F_{max}$ is the maximum fluorescence intensity, $F_0$ is the fluorescence intensity in the absence of DNA and $K_d$ is the dissociation constant.

RNA or DNA sequences utilised in the ITC as well as fluorescence studies are as follows:

**TAR RNA:** 5′ -GGCAGAUCUGAGCCUGGGAGCUCUCUGCC- 3′

**TAR DNA:** 5′ -GGCAGATCTGAGCCTGGGAGCTCTCTGCC- 3′

**TAR RNA w/b:** 5′ -GGCAGAGAGCCUGGGAGCUCUCUGCC- 3′

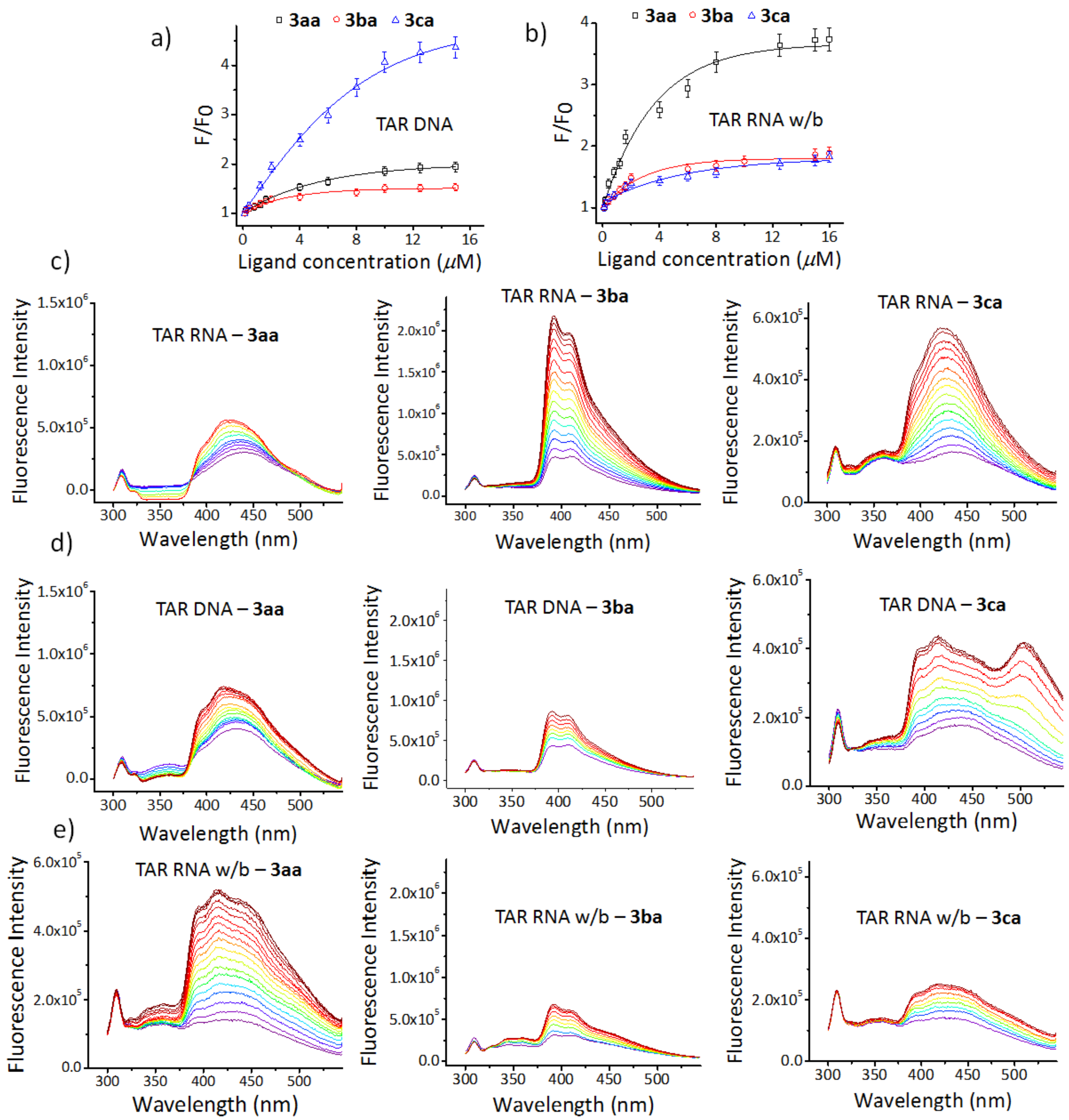


**Figure S6.** Fluorescence titration profiles of hit triazole ligands **3aa**, **3ba** and **3ca** (2 $\mu$M) with **(a)** TAR DNA (0-8 equiv.) and **(b)** TAR RNA w/b (0-8 equiv.). Fluorescence titration assays of ligands **3aa**, **3ba** and **3ca** with **(c)** TAR RNA, **(d)** TAR DNA and **(e)** TAR RNA w/b in 20mM Na cacodylate, 180 mM NaCl and 10 mM $MgCl_2$ buffer pH 7.4.

## 13.0 Fluorescence displacement study

Fluorescence spectra were measured in a Horiba JobinYvon Flurolog spectrofluorimeter FL3-11 at 25 °C in thermostated cell holder using 1 mm path length micro quartz cuvette with filtered buffer (20mM Na cacodylate, 180 mM NaCl and 10 mM $MgCl_2$ buffer pH 7.4). RNA or DNA was annealed as mentioned previously and mixed in 1:1 ratio with Tat peptide. Fluorescence displacement titration study was performed with successive addition of hit ligands. Final analysis of the data was carried out using Originpro 8.0 (OriginLab Corp.).

RNA and tat peptide sequences utilized in the fluorescence displacement studies are as follows:

**TAR RNA:** 5′ FAM-GGCAGAUCUGAGCCUGGGAGCUCUCUGCC- 3′

**Tat peptide:** $H_2N$-$_{57}$RRRQRRKKRGY$_{47}$-TAMRA

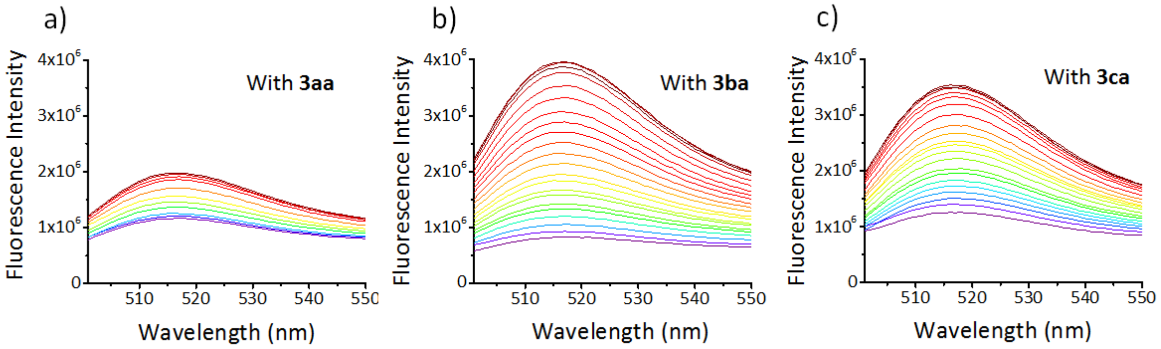


**Figure S7.** Fluorescence titration assay of Tat peptide (500 nM) displacement from TAR RNA (500 nM) upon titration with the hit triazole ligands **(a) 3aa**, **(b) 3ba** and **(c) 3ca** in 20 mM Na cacodylate, 180 mM NaCl and 10 mM $MgCl_2$ buffer pH 7.4.

## 14.0 Molecular docking

The energy minimized structures of ligands **3ba** and **3ca** were obtained with Gaussian 03 using density functional theory (DFT) analysis B3LYP/6-31+G(d) level. Docking studies were performed with the lowest energy conformer of ligands and TAR RNA (PDB ID: 1ANR) using the Auto-Dock 4.0 program. 30 docking calculations were performed using the Lamarckian genetic algorithm (LGA) with the default parameters from Auto-Dock 4.0 program. A maximum

of 25 million energy evaluations were applied for the experiment. The docked complex structures were imaged using Chimera 1.11.2 software.

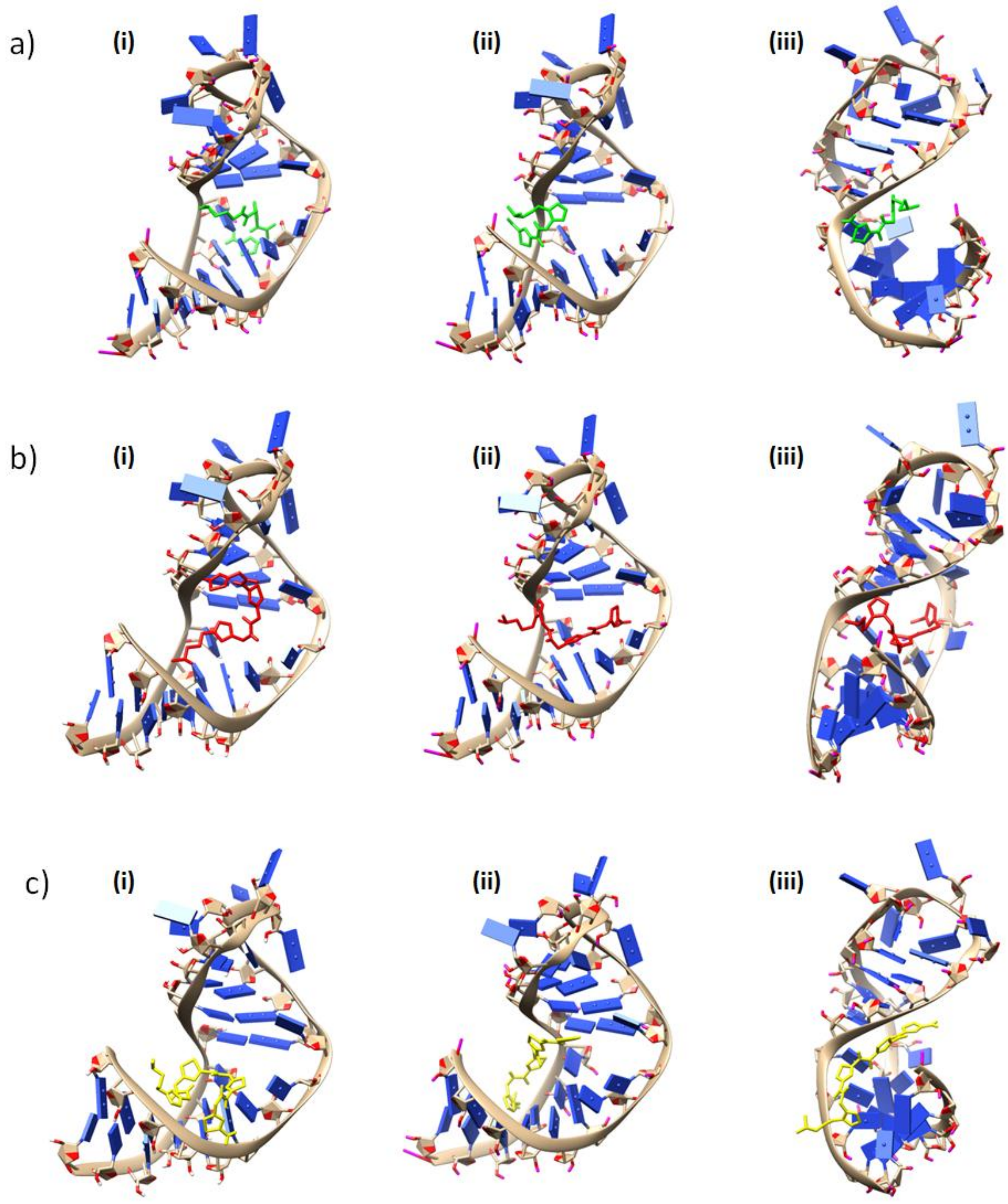


**Figure S8.** Molecular docking of ligands **(a) 3aa** (**(i)** *anti*-**3aa** and **(ii** & **iii**) *syn*-**3aa**), **(b) 3ba** (**(i)** *anti*-**3ba** and **(ii** & **iii**) *syn*-**3ba**) and **(c) 3ca** (**(i)** *anti*-**3ca** and **(ii** & **iii**) *syn*-**3ca**) with TAR RNA (PDB ID: 1ANR) using *AutoDock 4.0* program.

We have performed docking studies of the *syn* (1,5-triazole) isomers of **3aa**, **3ba** & **3ca** with TAR RNA to gain insights into the binding mode. As described in the manuscript (Figure 7), the thiazole ring of the *anti*-triazole lead **3ba** interacts with the bulge region of the TAR RNA and its water soluble chain interacts with the phosphate backbone of other strand making the interaction stable ($\Delta G$ = -9.12 Kcal mol$^{-1}$). On the other hand, molecular modeling shows that the binding interactions of the *syn*-**3ba** isomer is not feasible with TAR RNA ($\Delta G$ = -3.07 Kcal mol$^{-1}$) due to sterical hindrances. And it is also unable to interact with the bulge region of the TAR RNA (Figure S8).

For ligand **3ca,** the *anti* isomer attains a crescent shaped structure and involves in stacking interactions with the major groove of the TAR RNA ($\Delta G$ = -6.85 Kcal mol$^{-1}$) and TAR DNA. However, the *syn*-isomer shows less favorable interactions with TAR RNA ($\Delta G$ = -2.31 Kcal mol$^{-1}$) by stacking interactions. Moreover, both *anti*- and *syn*- isomer of ligand **3aa** shows non specific interactions with the TAR RNA with high $\Delta G$ values of 1.27 Kcal mol$^{-1}$ and 3.46 Kcal mol$^{-1}$ for *anti*-**3aa** and *syn*-**3aa**, respectively.
